# Phytocannabinoids Stimulate Rejuvenation and Prevent Cellular Senescence in Human Dermal Fibroblasts

**DOI:** 10.3390/cells11233939

**Published:** 2022-12-06

**Authors:** Marta Gerasymchuk, Gregory Ian Robinson, Alyssa Groves, Lucie Haselhorst, Sanjana Nandakumar, Cora Stahl, Olga Kovalchuk, Igor Kovalchuk

**Affiliations:** 1 Department of Biological Sciences, University of Lethbridge, Lethbridge, AB T1K 3M4, Canada; 2 Institute for Medical Nutrition Science, Universität zu Lübeck, 23562 Lübeck, Germany; 3 School of Biosciences and Technology, Vellore Institute of Technology, Vellore 632014, India; 4 Department of Medicine, Medical Sciences, and Nutrition, University of Aberdeen, King’s College, Aberdeen AB24 3FX, UK

**Keywords:** aging, skin, fibroblast, stress-induced premature senescence, cannabinoids, THC, CBD

## Abstract

In light of the increased popularity of phytocannabinoids (pCBs) and their appearance in beauty products without rigorous research on their rejuvenation efficacy, we decided to investigate the potential role of pCBs in skin rejuvenation. Utilizing healthy and stress-induced premature senescent (SIPS) CCD-1064Sk skin fibroblasts, the effects of pCBs on cellular viability, functional activity, metabolic function, and nuclear architecture were tested. Both delta-9-tetrahydrocannabinol (THC) and cannabidiol (CBD) within the range of 0.5 µM to 2.0 µM increased cell growth in a dose-dependent manner while significantly decreasing senescence as measured by beta-galactosidase activity. Utilizing a scratch assay, both THC and CBD (2.0 µM) significantly improved wound healing in both healthy and SIPS fibroblasts. THC and CBD altered nuclear architecture and mRNA levels of cell cycle regulators and genes involved in ECM production. Subsequently, we found ELN, Cyclin D1, PCNA, and BID protein levels altered by SIPS but ameliorated after pCBs exposure in human dermal fibroblasts. Lastly, we compared the efficacy of THC and CBD with common anti-aging nutrient signaling regulators in replicative senescent adult human dermal fibroblasts, CCD-1135Sk. Both THC and CBD were found to improve wound healing better than metformin, rapamycin, and triacetylresveratrol in replicative senescent CCD-1135Sk fibroblasts. Therefore, pCBs can be a valuable source of biologically active substances used in cosmetics, and more studies using clinical trials should be performed to confirm the efficacy of phytocannabinoids.

## 1. Introduction

*Cannabis sativa*, also known as marijuana, has been used for spiritual, medical, and industrial purposes for millennia. Cannabis-based products include clothing, bioplastics, and biofuel. However, most of the interest in cannabis is due to the active medical ingredients found within, which include over 100 phytocannabinoids (pCBs). The most abundant pCBs are Δ-9-tetrahydrocannabinol (THC) and cannabidiol (CBD) [1].

THC acts primarily as a weak partial agonist on cannabinoid (CB)1 and CB2 receptors modulating the endocannabinoid system. Well-known effects of THC include antiemetic, neuroprotective, anticancer, anti-inflammatory properties, and of course, psychoactivity [2,3]. CBD is another important cannabinoid and, in contrast to THC, has little direct affinity to CB1 and CB2 receptors. Rather, CBD is a negative allosteric modulator of CB1 with protean pharmacological effects on various other receptor systems, including vanilloid receptor 1, adenosine A2A, and non-receptor mechanisms [3]. CBD demonstrates neuroprotective, anti-inflammatory, antipsychotic, and antiseizure properties without THC-induced intoxication. Additionally, CBD improves blood circulation, provides antioxidant and antimicrobial activity, and helps with common skin disorders such as psoriasis, eczema, dermatitis, lupus, acne, and nail-patella syndrome [1]. Other minor pCBs, such as cannabigerol, cannabichromene, and Δ9-tetrahydrocannabivarin, also exhibit interesting pharmacological properties and usually modulate the effects of THC and/or CBD.

Although pCBs have been shown to be beneficial in treating psoriasis, eczema, and fibrosis [4,5,6,7], few studies have been performed on the efficacy of cannabis in cosmetology [1]. Regardless, many cannabis-containing beauty products have recently appeared on the market with many unproven claims on their benefits to the skin. Current but limited scientific data suggests cannabis might have beneficial properties for use in anti-aging or rejuvenation products; however, the extent of these benefits is unknown.

Aging is associated with the gradual damage of cells and tissues with subsequent accumulation of cell debris accompanied by inflammatory responses, general deterioration of function, and metabolic reprogramming. The first and most visible symptoms of aging are manifested by the skin’s appearance [8,9]. Although the epidermis is the visible layer, the dermis plays an integral role in delivering nutrients and structurally supporting the epidermis through a net of interconnected collagen and elastin fibers. Not only does this extracellular matrix (ECM) provide structural and tensile strength but plays a crucial role in skin hydration by binding water to collagen fibers found within the ECM. Due to genetic/epigenetic changes, environmental stressors, and successive replications, dermal fibroblasts can have inhibited mitochondrial function, alterations in nuclear architecture, and degradation of telomeres, resulting in dysregulation of gene expression in dermal fibroblasts that promote maintenance of the ECM. Over time, this causes degradation of the ECM, resulting in deterioration of the overlying cutaneous structure, causing the skin to become visually aged [8].

Studies have suggested CBs might also have a therapeutic effect on acne, dermatitis, pruritus, wound healing, and melanomas [2]. For instance, a recent study demonstrated that topical administration of palmitoylethanolamide (PEA), which stimulated anandamide (AEA) activity on CB1 receptors, reduced itch by 86.4% and was well-tolerated by patients [10]. Furthermore, results of a multicohort open-label trial showed that 32 patients with calciphylaxis, not caused by uremia, received topical CBD (3.75 mg/mL) and THC (<1 mg per day), which was applied to wound beds and peri-wound tissues. After 1 year of treatment, wound closure was achieved in 90% of cases [11]. Two different studies showed that topical application of CBD was associated with a reduction in blisters by at least 50%, with improved wound healing and decreased use of opioid analgesics in 6 pediatric patients aged 6 months to 10 years [12,13]. Moreover, lower doses of CBD were shown to be more effective than vitamins C and E in alleviating skin conditions such as acne [14,15].

As cannabinoids are lipophilic compounds that can easily pass-through biological barriers, such as skin, transdermal emulsions and creams containing phytocannabinoids could easily be utilized. Within the skin, transdermal application of pCBs could act upon the CB1/CB2 receptors or many other receptors, which are expressed in numerous skin cells, including epidermal keratinocytes, cutaneous nerves, sebaceous cells, eccrine sweat glands, mast cells, and macrophages. Furthermore, pCBs have been shown to provide beneficial anti-inflammatory, antioxidant, and immunomodulatory effects, both dependent and independent of CB1/2 receptors, which could be beneficial for cosmetic applications for anti-aging and rejuvenation of the skin [15].

Previously, we developed a stress-induced premature senescence (SIPS) phenotype that mimics typical replicative senescence while producing the senescence-associated secretory phenotype [16]. Utilizing this model, we compared nutrient signaling regulators (NSRs) alone and in combination with phytocannabinoids to determine if phytocannabinoids would be beneficial alone or/and potentiate the anti-aging effects of NSRs [17]. Although the addition of pCBs to NSRs was beneficial, THC or CBD alone appeared to have superior rejuvenation properties, growth and wound healing in senescent fibroblasts. Similarly, previous studies have demonstrated beneficial anti-aging effects on fibroblasts with the use of cannabis extracts which included phytocannabinoids; however, pure THC and CBD were not tested, and the compounds that induce these anti-aging effects were not identified [18,19,20,21]. In addition, some studies have shown CBD exposure on dermal fibroblasts prevented structural changes caused by psoriasis [22] and ameliorated proteome changes after UV damage [23]. There, however, remains a lack of knowledge on the anti-aging and rejuvenation effects of THC and CBD on healthy and aged human dermal fibroblasts. For this reason, we decided to thoroughly study the use of THC and CBD on cell growth, molecular markers, nuclear parameters, and wound healing to further study the potential use of phytocannabinoids in cosmetology using a human dermal fibroblast model.

## 2. Materials and Methods

### 2.1. Cell Culture and Maintenance

Normal human neonatal foreskin fibroblasts CCD-1064Sk (ATCC^®^ CRL-2076™) and normal human adult skin fibroblasts CCD-1135Sk (ATCC^®^ CRL-2691™) were purchased from American Type Culture Collection (Rockville, MD, USA). Dermal fibroblasts were grown as a monolayer in ISCOVE’s Modified Dulbecco’s Medium (IMDM) 1X (MULTICELL, Cat# 319-106-CL) containing 10% heat-inactivated Fetal Bovine Serum (Cat# 97068-085, VWR International LLC, Radnor, USA) and 1% Penicillin-Streptomycin (Cat# 450-201-EL, Wisent Inc., Sainte-Jean-Baptiste, QC, Canada) in a humidified incubator at 37 °C and 5% CO_2_.

### 2.2. Senescence-Associated Phenotype Modelling

Our previously established model of stress-induced premature senescence (SIPS) was utilized [17]. Briefly, dermal fibroblasts were incubated for 1 h with 25 µM of hydrogen peroxide solution (H_2_O_2_ dissolved in D-PBS) in a humidified atmosphere at 37 °C with 5% CO_2_. Following 1 h of exposure, the H_2_O_2_ solution was substituted with IMDM cell culture medium or designated potential anti-aging treatment.

### 2.3. Cannabinoid and Nutrient Signaling Regulators Treatments

Cell cultures were treated with THC (Cat# T4764, Sigma-Aldrich, Saint Louis, MO, USA) and CBD (Cat# C-045, Sigma-Aldrich, Saint Louis, MO, USA) at the following doses: 0.25 µM, 0.5 µM, 1 µM, 2 µM, 5 µM, 7.5 µM, and 10 µM. Cannabinoids were dissolved in dimethyl sulfoxide (DMSO), anhydrous (Cat# D12345, Life Technologies Corporation, Carlsbad, CA, USA). Following H_2_O_2_ exposure, cells were incubated with the designated treatment for 2 h daily for 5 days due to previous studies showing optimal benefits occurring after 1–2 h exposures [24,25]. Subsequently, the media was replaced without any additional treatments. Healthy fibroblasts (not exposed to H_2_O_2_) were treated with cannabinoids, as described above. In addition, CCD-1135Sk dermal fibroblasts were treated with three popular anti-aging drugs, rapamycin, metformin, and triacetylresveratrol (TRSV) at concentrations previously shown to be efficacious in anti-aging studies: 5 µM of rapamycin (Thermo Fisher Scientific, Waltham, MA, USA), 500 µM of metformin (Cedarlane, Toronto, ON, Canada), and 10 µM TRSV (VWR, Radnor, PA, USA) [18]. All anti-aging drugs were dissolved in DMSO and then dissolved in the media before being applied (*n* = 3 for each condition) for 2 h daily for 5 days.

### 2.4. Senescence-Associated β-Galactosidase Activity

Senescence-associated biomarker beta-galactosidase (β-Gal) was measured in triplicate in healthy and senescent dermal fibroblasts with a β-Gal Detection Kit (Cat# AB176721, Abcam, Cambridge, UK) according to the manufacturer’s instructions [17]. The fluorescence intensity of the fluorogenic fluorescein digalactoside (FDG) product was measured through a microplate reader at Ex490/Em525 nm (BMG LABTECH, Cary, NC, USA). β-galactosidase activity was not normalized to the cell number.

### 2.5. Cell Viability/Cytotoxicity Assays

Cellular viability of CCD-1064Sk human skin fibroblasts was assessed by the methyl thiazole tetrazolium (MTT) reduction assay, crystal violet (CV), and neutral red (NR) staining assays as previously described [26,27]. MTT (3-[4,5-dimethylthiazol-2-yl]-2,5-diphenyltetrazolium bromide; thiazolyl blue) is based on the reductive activity of the mitochondrial succinic dehydrogenase enzyme. MTT is a sensitive and reliable indicator of cell metabolic activity and was performed using the cell proliferation kit I (Cat#11465007001, Roche, ON, Canada) per the manufacturer’s instructions. NR staining demonstrates the ability of viable cells to incorporate and bind neutral red dye within the lysosomes [26]. The CV dye was used to assess the viability of cultured fibroblasts and performed according to previously published methods [18,26]. Cells with less crystal violet staining typically lose their adherence and undergo cell death [26]. Stained dermal fibroblasts were photographed using a Zeiss Ob-server Z1 epifluorescence microscope with AxioVision Rel 4.8 software (Carl Zeiss Canada Ltd., Toronto, ON, Canada).

### 2.6. Protein Extraction and Western Blot Analysis

Protein extraction was performed with 100–150 μL RIPA lysis buffer. Subsequently, 50 μg of each sample was analyzed by sodium dodecyl sulfate-polyacrylamide gel electrophoresis. Primary and secondary antibodies utilized are presented in Supplementary Table S1. The antigen-antibody complex was detected with the ECL Prime Western Blotting System (Cat#GERPN2232, GE Healthcare, Chicago, IL, USA). Protein bands were visualized by the FluorChem HD2 Imaging System (Cell Biosciences, Santa Clara, CA, USA). Protein band intensity was quantified with Image J and normalized to GAPDH. Membranes were stripped and re-probed, as indicated in the supplementary figures.

### 2.7. Reverse Transcription Polymerase Chain Reaction (RT-PCR)

Total RNA was isolated using the TRIzol^®^ Reagent (Invitrogen, Carlsbad, CA, USA) and converted to cDNA using iScriptTM Select cDNA synthesis kit (Cat# 1708897, BioRad, Hercules, CA, USA) according to the manufacturer’s instruction. Quantitative real-time PCR (qPCR) was performed with SsoFastTM EvaGreen^®^ Supermix (Cat# 1725202, Bio-Rad) in a C1000TM Thermo Cycler equipped with a CFX96 Touch™ Real-Time PCR Detection System (BioRad). The PrimerQuest™ Tool platform was used for primer design (Table S2). Expression levels of target genes were normalized to *GAPDH* in each group. The comparative CT method (ΔΔCt method) was used to calculate relative fold expression levels using the BioRad Software (CFX Manager) [28].

### 2.8. Wound-Healing Assay

CCD-1064Sk and CCD-1135Sk fibroblasts were grown in the monolayer to >90% confluence in 24-well plates. Consistently shaped wounds/scratches were made using a sterile 10 mL pipette tip across each well, creating a cell-free area of approximately 0.4–0.5 mm in width. The culture medium was then immediately removed, and fibroblasts were washed twice with PBS to remove loose cells. After that, the removed medium was replaced with a designated treatment or IMDM cell culture medium. Wound closure was monitored by collecting digitized images at 1 h, 24 h, 48 h, and 72 h intervals after the scratch. Images of the healing process were taken using the Infinity3 camera in the OLYMPUS CKX41 microscope. The remaining wounded area and the scratch width at seven different points per image were measured and analyzed with ImageJ (IJ 1.46r) software. Data has been presented as the percentage of an unhealed wound, i.e., the percentage by which the original scratch width has decreased for each given time point. All scratch assays were performed in sextuplicate.

### 2.9. Immunocytochemistry, Microscopy, and QuPath Analysis

The immunocytochemistry, microscopy, and QuPath analysis were performed as previously described [17,18] utilizing 4′,6-diamidino-2-phenylindole (DAPI) to stain nuclei (Thermo Fisher Scientific, Cat# D1306) according to the manufacturer’s instructions. Photographs of the DAPI-stained nuclei were taken using a Zeiss Observer Z1 epifluorescence microscope with Axio-Vision Rel 4.8 software.

Utilizing the cell identification function, QuPath 0.2 software [29] determined area (μm^2^), perimeter (μm), circularity, eccentricity, and max and min calipers (μm) for each visually verified nucleus.

### 2.10. Statistical Analysis

GraphPad Prism 9.3.1 (GraphPad Software, San Diego, CA, USA) was used for statistical analyses and graphic generation. Data are presented as means with standard error of the mean (SEM), except where noted. A one-way ANOVA test followed by a Dunnett’s compared to the vehicle or Tukey’s post-hoc multiple comparison tests were performed for statistical analysis. The following significance (*p*) scale was indicated within the figures using: * *p* < 0.05; ** *p* < 0.01; *** *p* < 0.001; and **** *p* < 0.0001.

## 3. Results

### 3.1. Optimizing Phytocannabinoid Dosing on Human Dermal Fibroblasts

To determine the optimal concentration of cannabinoids to use for testing for potential anti-aging effects, fibroblasts were exposed to a range of doses to determine if any cytotoxic effects were seen. Healthy fibroblasts were treated with THC and CBD dissolved in DMSO at the following doses: 0.25 µM, 0.5 µM, 1 µM, 2 µM, 5 µM, 7.5 µM, and 10 µM (Figure S1). CCD-1064Sk fibroblasts were incubated in THC or CBD for 2 h daily for 5 days since therapeutic treatments of fibroblasts and skin have optimal responses after 1–2 h.

Phase-contrast imaging was used to characterize qualitative changes in dermal fibroblasts (Figure S1). Our observations showed that low doses of THC and CBD (i.e., 0.25 µM, 0.5 µM, 1 µM) have a cytostatic effect as the number of cells in the culture remained almost unchanged. Morphologically, fibroblasts treated with low concentrations of cannabinoids were similar to the untreated fibroblasts. At the same time, a cytotoxic effect was observed in response to 7.5 and 10 µM concentrations of CBD and 10 µM THC. There was an obvious reduction in cell quantity after five days of cannabinoid treatments. The cellular architecture was drastically affected: fibroblasts were decreased in size with a coin-like round shape or visible degradation surrounded by remnants of debris (Figure S1).

**Figure 1:**
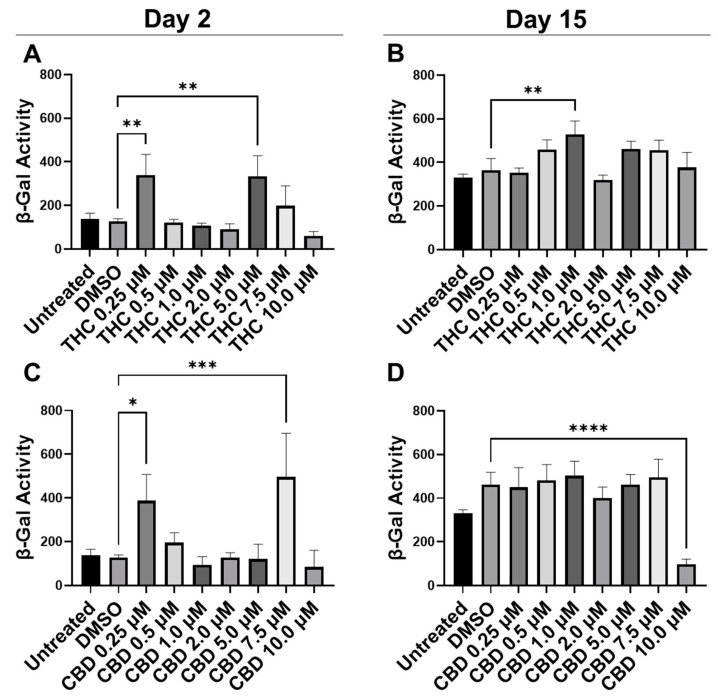
β-Gal activity in CCD-1064Sk human dermal fibroblasts (PDL 48) exposed to different concentrations of THC and CBD. (**A**) β-Gal activity after day 2 of THC exposure. (**B**) β-Gal activity after 15 days of THC exposure. (**C**) β-Gal activity after day 2 of CBD exposure. (**D**) β-Gal activity after 15 days of CBD exposure. Data were analyzed with an ANOVA test followed by a Dunnett’s post-hoc multiple comparison test compared to the DMSO vehicle. Bars represent mean ± SEM, *n* = 3. Significance is indicated using the following scale: * *p* < 0.05, ** *p* < 0.01, *** *p* < 0.001, **** *p* < 0.0001. CBD, cannabidiol; DMSO, dimethyl sulfoxide (vehicle); THC, delta-9-tetrahydrocannabinol.

To quantify potential maladaptive responses to phytocannabinoids, CCD-1064Sk fibroblasts in replicative senescence (RS) at population doubling level (PDL) 48 were treated with 0.25 µM, 0.5 µM, 1 µM, 2 µM, 5 µM, 7.5 µM, and 10 µM concentrations of THC and CBD for 15 days, 2 h daily (Figure 1). Treatment length was chosen due to the previous studies showing optimal benefits of therapeutics occurring after 1–2 h exposure [24,25]. The β-Gal assay was used on day 2 and day 15 to determine if pCBs would alter senescence-associated β-Gal activity [30]. Two days after the start of pCBs treatment, β-Gal activity was significantly higher in the fibroblasts treated with 0.25 µM and 5.0 µM of THC (*p* < 0.01, Figure 1A) and 0.25 µM (*p* < 0.05) and 7.5 µM of CBD (*p* < 0.001, Figure 1C) compared to the vehicle (DMSO). Following 15 days of treatment with pCBs, β-Gal activity in fibroblasts compared to the vehicle was elevated after 1 µM of THC exposure (*p* < 0.01, Figure 1B) and was significantly lower with 10 µM of CBD exposure (*p* > 0.0001, Figure 1D), while similar levels of β-Gal were seen at the 2 µM THC and 2 µM CBD in RS fibroblasts (Figure 1B,D). While 10 µM of CBD did decrease β-Gal activity, which might be seen as beneficial, this is likely due to decreased cell number by the severe cytotoxic effect seen via phase-contrast microscopy at higher concentrations of CBD (Figure S1).

### 3.2. Phytocannabinoids Enhance Cellular Viability in SIPS Fibroblasts

Since 2.0 µM of THC and 2.0 µM of CBD did not have cytotoxic effects and preserved cellular morphology (Figure S1), we decided to further study these concentrations. Simultaneously, we decided to test 0.5 µM of THC and CBD as they did not appear to be cytotoxic or induce senescence after either 2 or 15 days of exposure (Figure S1 and Figure 1).

**Figure 2:**
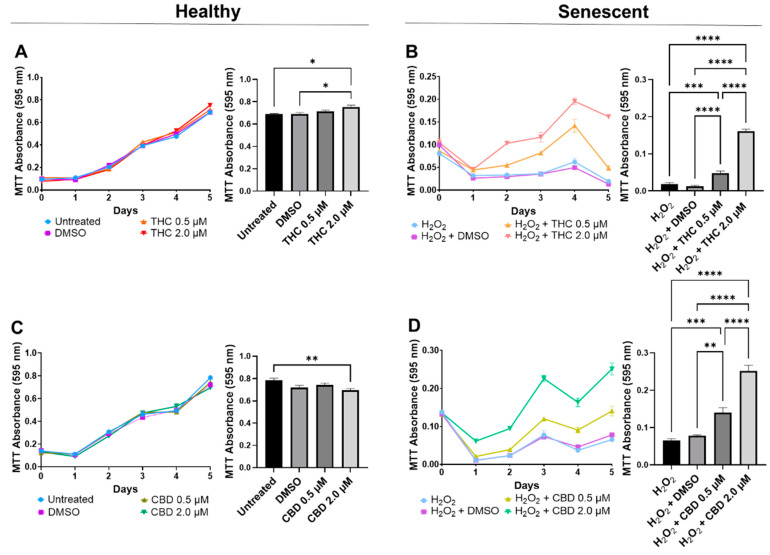
Cell viability of CCD-1064Sk human dermal fibroblasts (PDL 24) treated with THC or CBD at concentrations of 0, 0.5, or 2 µM. (**A**) Healthy fibroblasts treated with 0, 0.5, or 2 µM of THC. (**B**) SIPS fibroblasts treated with 0, 0.5, or 2 µM of THC. (**C**) Healthy fibroblasts treated with 0, 0.5, or 2 µM of CBD. (**D**) SIPS fibroblasts treated with 0, 0.5, or 2 µM of CBD. Cell viability was quantified by MTT assay. Data were analyzed with an ANOVA test followed by Tukey’s post-hoc multiple comparison test. Bars represent mean ± SEM, *n* = 3. Significance is indicated using the following scale: * *p* < 0.05, ** *p* < 0.01, *** *p* < 0.001, **** *p* < 0.0001. CBD, cannabidiol; DMSO, dimethyl sulfoxide (vehicle); THC, delta-9-tetrahydrocannabinol.

In healthy fibroblasts, cellular viability measured by MTT was similar between the untreated, DMSO, and 0.5 µM of either THC or CBD (Figure 2A,C), suggesting no difference after pCBs treatments. In contrast, 2 µM of THC increased cell viability compared to the vehicle (*p* < 0.05, Figure 2A), while 2 µM of CBD decreased cell viability compared to the untreated (*p* < 0.01) but not the vehicle (*p* > 0.05, Figure 2C). Together, this data may suggest 2 µM of THC may increase cell viability of healthy dermal fibroblasts, while CBD showed no adverse or beneficial effect (Figure 2). Conversely, in our SIPS model, both THC (*p* < 0.0001) and CBD (*p* < 0.01) improved cellular viability compared to the vehicle and displayed a dose-dependent response, with the 2 µM dose of either THC or CBD having significantly higher cell viability than the corresponding 0.5 µM dose (*p* < 0.0001, Figure 2B,D). Based on these findings, the 2 µM concentration of CBD and THC was chosen as the optimal concentration for all subsequent experiments.

**Figure 3:**
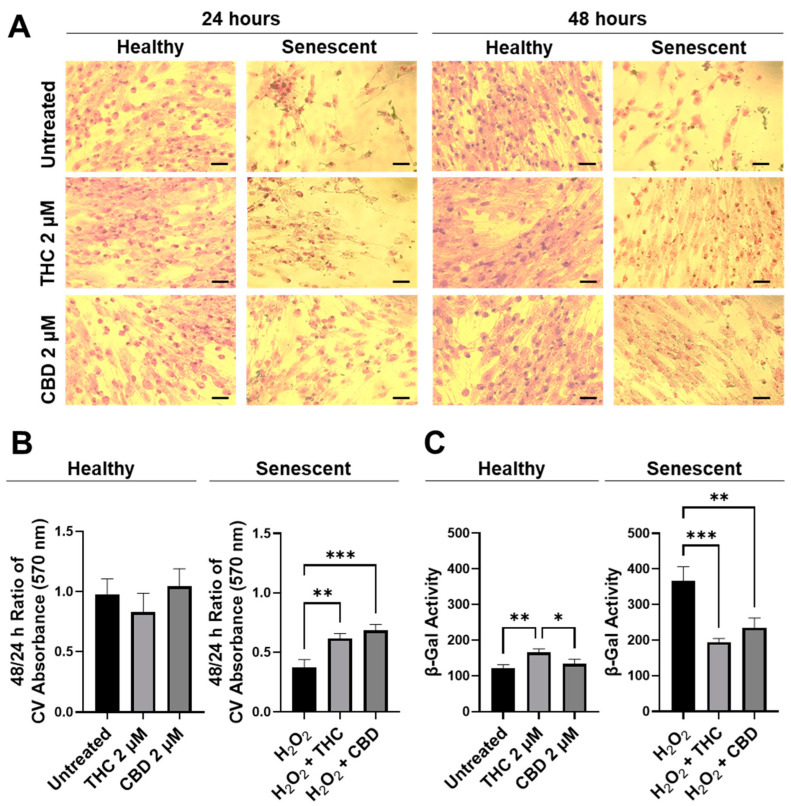
CV-stained fibroblasts, β-Gal activity, and 48/24 h CV assay ratio in healthy and SIPS CCD-1064Sk human dermal fibroblasts (PDL 24) exposed to 2 µM of THC or CBD. (**A**) Images of healthy and senescent fibroblasts stained with CV after 24 or 48 h of treatment. Scale = 50 μm. (**B**) The 48-to-24-h ratio of CV assay performed after treatment with THC or CBD exposure in healthy and SIPS fibroblasts. (**C**) β-Gal activity after 5 days of treatment with THC or CBD in healthy and SIPS fibroblasts. Data were analyzed with an ANOVA test followed by Tukey’s post-hoc multiple comparison test. Bars represent mean ± SEM, *n* = 3. Significance is indicated using the following scale: * *p* < 0.05, ** *p* < 0.01, *** *p* < 0.001. CBD, cannabidiol; CV, crystal violet; DMSO, dimethyl sulfoxide (vehicle); SIPS, stress-induced premature senescence; THC, delta-9-tetrahydrocannabinol.

Next, the efficacy of THC and CBD was compared by performing crystal violet staining (Figure 3A) and 48/24-hr CV assays (Figure 3B). The CV assay measures the number of healthy cells that are attached to the substrate after treatment. No significant changes were seen in healthy cells (Figure 3B); however, in SIPS fibroblasts, an increased ratio of CV-positive cells was detected following THC (*p* < 0.01) and CBD (*p* < 0.001) exposure compared to the H_2_O_2_ group (Figure 3B) demonstrating phytocannabinoids increase cell viability. Subsequently, senescence-associated β-Gal activity was measured in healthy and SIPS fibroblasts. Surprisingly, we found THC increased β-Gal activity compared to the untreated group (*p* < 0.01) and to the CBD group, suggesting an increase in senescence in healthy cells (*p* < 0.05, Figure 3). In contrast, β-Gal activity was significantly reduced by both THC (*p* < 0.001) and CBD (*p* < 0.01) compared to the vehicle in SIPS fibroblasts, suggesting a decrease in senescence (Figure 3C). This finding may suggest THC minimally but significantly increases senescence biomarkers in healthy fibroblasts.

To better visualize morphological results, healthy and senescent skin fibroblasts were stained with crystal violet (binds to proteins and DNA) and neutral red (accumulates in the lysosomes of viable cells). Significant structural alterations were detected in senescent cells as compared to healthy ones or those treated with pCBs (Figure S2). Prematurely aged cells treated with THC and CBD exerted mild modifications of shape and size, unlike aged fibroblasts. Overall, our results show that pCBs treatment exerts a significant positive effect on the preservation of senescent cell viability with minimal effects on healthy fibroblasts (Figure 3 and Figure S2).

### 3.3. Phytocannabinoids Improve Wound Healing of Human Dermal Fibroblasts

One of the most prominent roles of human dermal fibroblasts is maintaining healthy skin by repairing and regenerating damaged tissues. To test the regenerative ability of dermal fibroblasts, we performed a wound healing assay (WHA), also known as a scratch assay, on healthy and SIPS dermal fibroblast cells in the presence of THC, CBD, or vehicle.

**Figure 4:**
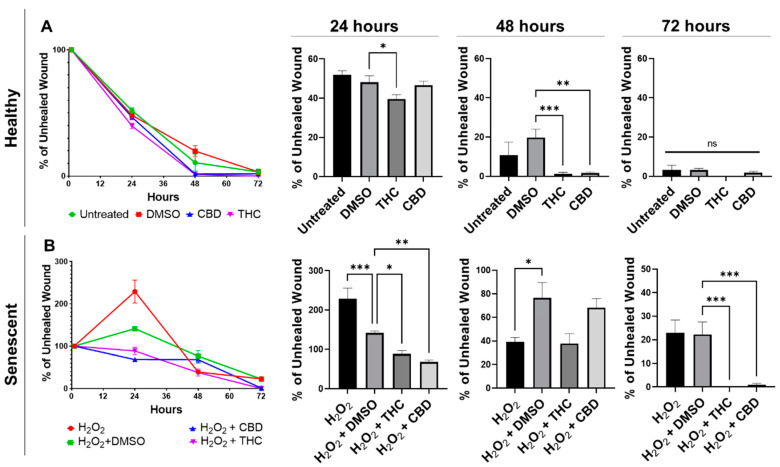
Phytocannabinoids improve wound healing in healthy and SIPS CCD-1064Sk (PDL 24) human dermal fibroblasts. (**A**) Percentage of the unhealed wounds at 24, 48, and 72 h after the scratch assay was performed with healthy dermal fibroblasts exposed to 2 µM of THC or CBD. (**B**) Percentage of the unhealed wounds at 24, 48, and 72 h after the scratch assay was performed with SIPS dermal fibroblasts exposed to 2 µM of THC or CBD. Data were analyzed with an ANOVA test followed by a Dunnett’s post-hoc multiple comparison test compared to the vehicle. Bars represent mean ± SEM, *n* = 6. Significance is indicated using the following scale: * *p* < 0.05, ** *p* < 0.01, *** *p* < 0.001. CBD, cannabidiol; DMSO, dimethyl sulfoxide (vehicle); SIPS, stress-induced premature senescence; THC, delta-9-tetrahydrocannabinol.

Wound healing in healthy fibroblasts was accelerated by both THC and CBD (Figure 4A). Healthy fibroblasts treated with THC had a significantly smaller percentage of unhealed wounds compared to the vehicle after 24 h (*p* < 0.05) and after 48 h (*p* < 0.001, Figure 4A). By 48 h, THC-treated healthy fibroblasts were essentially fully healed. In contrast, wounds of healthy CBD-treated fibroblasts were not significantly better after 24 h, but by 48 h, the wound area was essentially fully covered, and the percentage of the unhealed wound was significantly smaller than vehicle (*p* < 0.01, Figure 4A). By 72 h, scratches to healthy fibroblasts were fully recovered, and no significant differences were seen between any groups in healthy fibroblasts (Figure 4A).

Similar to healthy fibroblasts, phytocannabinoids improved wound healing in SIPS fibroblasts. Interestingly, the percentage of wound healing was exacerbated, and the wound grew in hydrogen peroxide treated fibroblasts after 24 h, while both THC (*p* < 0.05) and CBD (*p* < 0.01) significantly decreased the percentage of unhealed wounds (Figure 4B). Although no significant differences were seen between the pCBs groups and the vehicle, by 72 h, both THC and CBD had a significantly lower percentage of unhealed wounds compared to the vehicle (*p* < 0.001, Figure 4B). At 72 h, wounds induced in THC- and CBD-treated SIPS fibroblasts were completely healed, while SIPS fibroblasts that were not treated with pCBs had ~20% of the wound still unhealed (Figure 4B).

Due to the changes caused by pCBs and the ability of pCBs to increase wound healing speed in healthy and SIPS fibroblasts, we decided to compare pCBs to common anti-aging nutrient signaling regulators (NSRs). To best capture responses that would be seen in aged skin, we decided to utilize CCD-1135Sk dermal fibroblasts, which are derived from adult human skin, which we passaged to replicative senescence (PDL 40). We compared wound healing in response to THC, CBD, metformin, triacetylresveratrol, rapamycin, and the vehicle. We did not find any significant differences after 24 h; however, after 48 h, THC demonstrated improved wound healing compared to the vehicle (*p* < 0.05), Metformin (*p* < 0.0001), TRSV (*p* = 0.18), and rapamycin (*p* < 0.05, Figure S3). Similar to CCD-1064Sk dermal fibroblasts, THC was able to close the wound after 48 h (Figure 4A and Figure S3). After 72 h, THC still demonstrated a significantly lower percentage of unhealed wounds compared to metformin (*p* < 0.05), triacetylresveratrol (*p* < 0.01), rapamycin (*p* < 0.01), and the mixed treatment (*p* < 0.001, Figure S3). In addition, CBD demonstrated improved wound healing compared to rapamycin (*p* < 0.01) and the mixed group (*p* < 0.05) after 72 h, but not to any NSR after 48 h. However, at all time points, CBD did not appear to improve wound healing better than the vehicle (Figure S3).

### 3.4. Phytocannabinoids Alter Nuclear Morphology of Human Dermal Fibroblasts

Recently, many studies have shown abnormalities in nuclear morphology are commonly seen as hallmarks of aging and senescence. Nuclei can enlarge, become lobulated, fragment, and have invaginations, which can all alter heterochromatin structure resulting in altered gene expression. Previously, we have shown changes in the nuclear architecture of senescent versus healthy CCD-1064Sk human dermal fibroblasts [17]. Nuclei were enlarged and varied in size and shape in aged fibroblasts compared to healthy fibroblasts [17]. Therefore, we decided to determine if phytocannabinoids can ameliorate these changes induced by aging.

**Figure 5:**
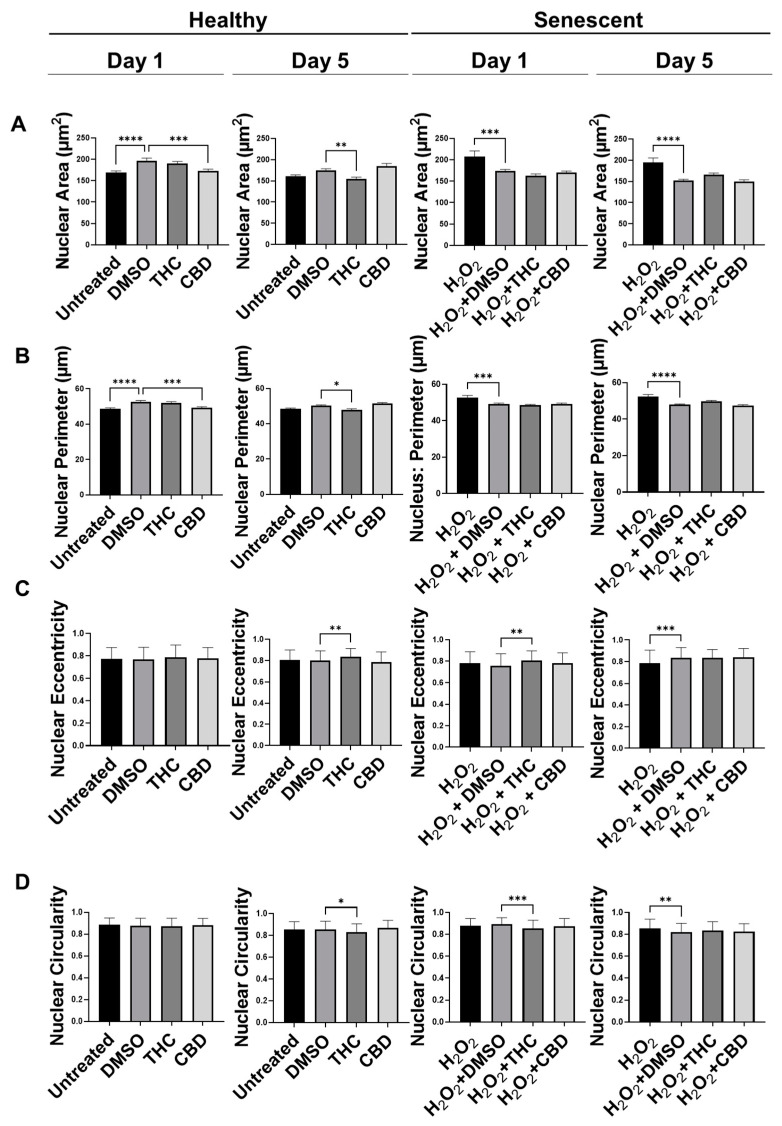
Nuclear parameters of healthy and SIPS DAPI-stained CCD-1064Sk dermal fibroblasts (PDL 24) exposed to phytocannabinoids. (**A**) Nuclear area, (**B**) nuclear perimeter, (**C**) nuclear eccentricity, and (**D**) nuclear circularity of healthy SIPS fibroblasts exposed to 2 µM of THC or CBD. Data were analyzed with an ANOVA test followed by a Dunnett’s post-hoc multiple comparison test compared to the vehicle (*n* = 76–186). Bars represent mean ± SEM. Significance is indicated using the following scale: * *p* < 0.05, ** *p* < 0.01, *** *p* < 0.001, **** *p* < 0.0001. CBD, cannabidiol; DMSO, dimethyl sulfoxide (vehicle); SIPS, stress-induced premature senescence; THC, delta-9-tetrahydrocannabinol.

Similar to our previous results, nuclei of fibroblasts exposed to the H_2_O_2_ displayed more elongated morphology than untreated cells in the CCD-1064Sk cell line, while some retained round architecture (Figure S4). Gigantic nuclei with irregular shapes were detected in SIPS fibroblasts (Figure S4). The nuclear architecture of dermal fibroblasts treated with both CBD and THC remained unchanged almost entirely in both healthy and SIPS fibroblasts (Figure S4 and Figure 5). The only noted change was decreased nuclei area and perimeter after CBD application in healthy fibroblasts on day one compared to the vehicle (*p* < 0.001), however, these changes were reverted by day 5 (Figure 5A,B). In contrast, THC dramatically affected nuclear architecture. In healthy fibroblasts, the area was decreased (*p* < 0.01), the perimeter was decreased (*p* < 0.05), eccentricity was increased (*p* < 0.01), and circularity was decreased (*p* < 0.05) after 5 days of THC treatment compared to the vehicle. In SIPS fibroblasts, THC increased eccentricity (*p* < 0.01, Figure 5C) and decreased circularity (*p* < 0.001, Figure 5D) after one day of THC treatment compared to the vehicle. Due to these alterations found in nuclear structure induced by phytocannabinoids, we decided to study the expression of age-related genes that are important for dermal aging.

### 3.5. Protective Effects of Phytocannabinoids on the Expression of Age-Related Genes Involved in Extracellular Matrix Maintenance

Fibroblasts are the main producers of extracellular matrix components (ECM), including collagen, elastin, and hyaluronan, which are necessary to maintain the proper functioning of the skin. Previously, we have shown significant deterioration of ECM elements in senescent cells in our SIPS model [17]. Subsequently, it was hypothesized that phytocannabinoids have rejuvenation properties by restoring collagen, elastin, and hyaluronic acid production.

**Figure 6:**
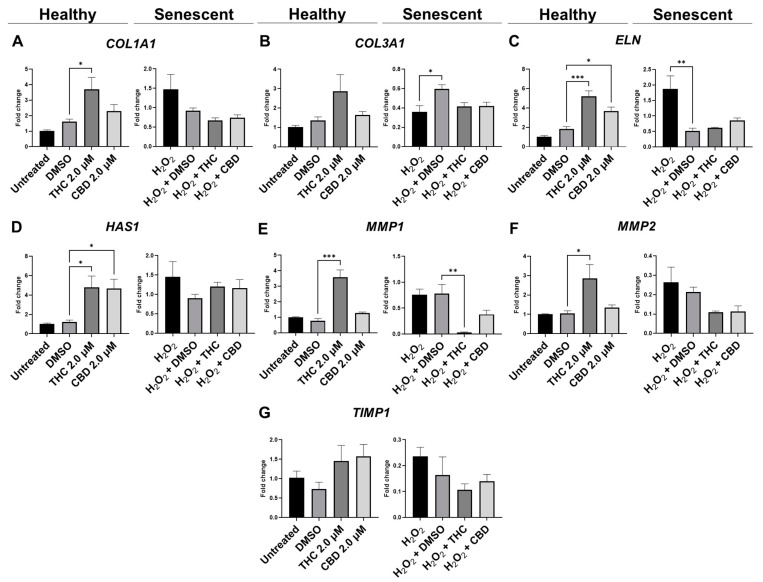
Effects of phytocannabinoids on mRNA levels of genes involved in extracellular matrix maintenance in CCD-1064Sk human dermal fibroblasts. mRNA levels were presented as a ratio of the target gene relative to *GAPDH* for expression of (**A**) *COL1A1*, (**B**) *COL3A1*, (**C**) *ELN*, (**D**) *HAS1,* (**E**) *MMP1*, (**F**) *MMP2*, and (**G**) *TIMP1*. Data were analyzed with an ANOVA test followed by a Dunnett’s post-hoc multiple comparison test compared to the vehicle. Bars represent mean ± SEM, *n* = 3. Significance is indicated using the following scale: * *p* < 0.05, ** *p* < 0.01, *** *p* < 0.001. CBD, cannabidiol; DMSO, dimethyl sulfoxide (vehicle); THC, delta-9-tetrahydrocannabinol.

qPCR analysis showed mRNA levels of *type 1 collagen* (*COL1A1*) were significantly upregulated during THC exposure compared to the vehicle in healthy fibroblasts but not in SIPS fibroblasts (*p* < 0.05, Figure 6A). mRNA levels of *elastin* (*ELN*) were significantly upregulated during THC (*p* < 0.001) or CBD exposure (*p* < 0.05) compared to the vehicle in healthy fibroblasts but not in SIPS fibroblasts (Figure 6C). In addition, THC and CBD exposure (*p* < 0.05) significantly upregulated *hyaluronan synthase* (*HAS1*) mRNA levels compared to the vehicle in healthy fibroblasts but not in SIPS fibroblasts (Figure 6D). The collagenase *matrix metalloproteinase-1* (*MMP1*) was found to be upregulated in healthy fibroblasts (*p* < 0.001) while downregulated in SIPS fibroblasts (*p* < 0.01), compared to their vehicles (Figure 6E). Furthermore, the gelatinase *matrix metalloproteinase-2* (*MMP2*) levels were upregulated in THC-exposed healthy fibroblasts compared to the controls (*p* < 0.05, Figure 6F) but not in SIPS fibroblasts. No change was seen in mRNA levels of *tissue inhibitor of metalloproteinases* (*TIMP1*) across any treatment (Figure 6F). Together, this demonstrates phytocannabinoids, specifically THC, can upregulate the genes that help produce collagen, elastin, and hyaluronan, which are the main structural components of the ECM, and increase the turnover of the ECM by increased *MMP1* and *MMP2* expression.

**Figure 7:**
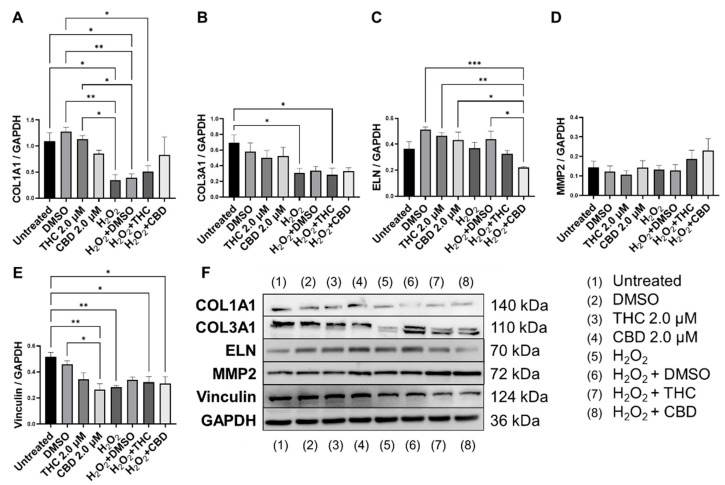
Effects of phytocannabinoids on extracellular matrix proteins in CCD-1064Sk human dermal fibroblasts (PDL 24). Relative densitometry was presented as a ratio of the target protein relative to GAPDH for expression of (**A**) COL1A1, (**B**) COL3A1, (**C**) ELN, (**D**) MMP2, and (**E**) Vinculin, (**F**) representative images blots with each protein detected. Original membranes can be seen in Figures S5–S10. Data were analyzed with an ANOVA test followed by Tukey’s post-hoc multiple comparison test. Bars represent mean ± SEM, *n* = 3. Significance is indicated using the following scale: * *p* < 0.05, ** *p* < 0.01, *** *p* < 0.001. CBD, cannabidiol; DMSO, dimethyl sulfoxide (vehicle); THC, delta-9-tetrahydrocannabinol.

Next, we performed Western blots on multiple proteins associated with ECM maintenance. As expected, our SIPS model, induced by hydrogen peroxide, caused a significant decrease in COL1A1 expression (*p* < 0.05, Figure 7A,B) and COL3A1 expression (*p* < 0.05, Figure 7B) compared to the untreated group. CBD treatment in SIPS fibroblasts appeared to upregulate COL1A1 expression compared to the vehicle (*p* = N.S., Figure 7A), while ELN expression was significantly downregulated compared to the vehicle (*p* < 0.05, Figure 7C) and similar to levels seen in the untreated group. Even though type 3 collagen is one of the most abundant proteins found in the skin ECM, no differences were seen between senescent groups (Figure 7B). Importantly, MMP2, known to degrade ECM constituents, was slightly upregulated following CBD (*p =* N.S., Figure 7D) application in the healthy group, while it tended to increase in senescent cells treated with pCBs (*p =* N.S., Figure 7D). Surprisingly, vinculin was downregulated by CBD in healthy fibroblasts compared to the vehicle (*p* < 0.05) and the untreated (*p* < 0.01, Figure 7E) group. Despite CBD and THC induced differences in ECM gene expression, pCBs did not significantly alter ECM protein abundance compared to the associated vehicles (Figure 7).

### 3.6. Effects of Phytocannabinoids on Cell Cycle Regulators

**Figure 8:**
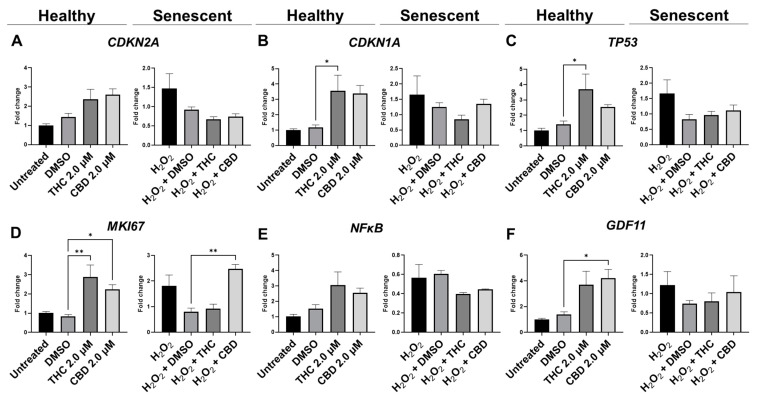
Effects of phytocannabinoids on mRNA levels of cell cycle regulators in CCD-1064Sk human dermal fibroblasts (PDL 24). mRNA levels were presented as a ratio of the target gene relative to *GAPDH* for expression of (**A**) *CDKN2A*, (**B**) *CDKN1A*, (**C**) *TP53*, (**D**) *MKI67,* (**E**) *NF-κB*, and (**F**) *GDF11*. Data were analyzed with an ANOVA test followed by a Dunnett’s post-hoc multiple comparison test compared to the vehicle. Bars represent mean ± SEM, *n* = 3. Significance is indicated using the following scale: * *p* < 0.05, ** *p* < 0.01. CBD, cannabidiol; DMSO, dimethyl sulfoxide (vehicle); THC, delta-9-tetrahydrocannabinol.

**Figure 9:**
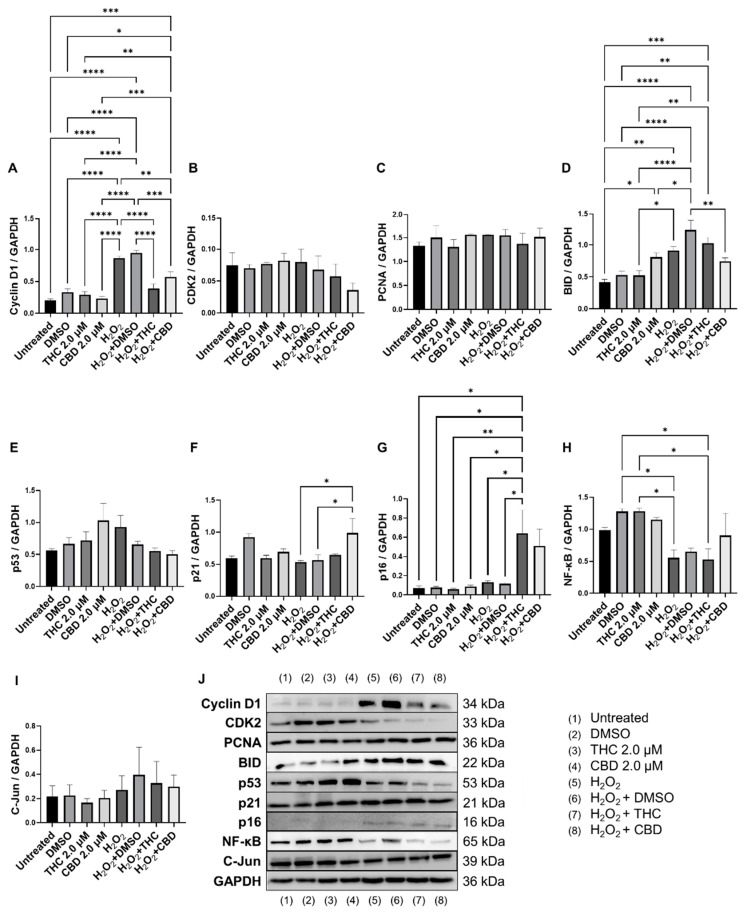
Effects of phytocannabinoids on cell cycle regulators in CCD-1064Sk human dermal fibroblasts (PDL 24). Relative densitometry was presented as a ratio of the target protein relative to GAPDH for expression of (**A**) Cyclin D1, (**B**) CDK2, (**C**) PCNA, (**D**) BID, (**E**) p53, (**F**) p21, (**G**) p16, (**H**) NF-κB, and (**I**) C-Jun. (**J**) representative images blots with each protein detected. Original membranes can be seen in Figures S11–S20. Data were analyzed with an ANOVA test followed by Tukey’s post-hoc multiple comparison test. Bars represent mean ± SEM, *n* = 3. Significance is indicated using the following scale: * *p* < 0.05, ** *p* < 0.01, *** *p* < 0.001, **** *p* < 0.0001. CBD, cannabidiol; DMSO, dimethyl sulfoxide (vehicle); THC, delta-9-tetrahydrocannabinol.

Since cell cycle regulators are known to be altered in senescence and aging, we analyzed mRNA levels (Figure 8) and protein levels of important cell cycle regulators (Figure 9). Cyclin D1 is one of the most commonly altered cell cycle regulators in aging and senescence. Cyclin D1 binds to cyclin-dependent kinases (CDK) to form a complex and promote passage from the G1 phase to the beginning of the S phase [31]. In addition, cyclin D1 acts independently of CDKs to regulate cell proliferation, growth, and differentiation [32]. In aging and senescence, cyclin D1 is overexpressed, but cell proliferation is inhibited downstream, usually by CDK inhibitors [32,33,34]. Thus, we measured cyclin D1 levels (Figure 9A). We found cyclin D1 was upregulated 4-fold in hydrogen peroxide exposed fibroblasts (SIPS) compared to the untreated group (*p* < 0.0001, Figure 9A). Simultaneously, in SIPS fibroblasts, both THC (*p* < 0.0001) and CBD (*p* < 0.001, Figure 9A) significantly decreased cyclin D1 levels compared to the SIPS vehicle. Not only does this reconfirm that our model accurately demonstrates SIPS through our Western blot data, but also shows phytocannabinoids can ameliorate cyclin D1 levels. Next, we measured CDK2 protein levels and found no significant changes between any groups, although SIPS fibroblasts had trended lower in the expression of CDK2 after pCBs treatment than healthy fibroblasts (*p* = N.S., Figure 9B).

Due to changes in cyclin D1 protein expression, we decided to look at other known markers of proliferation. *MKI67* encodes a protein that is a known marker associated with cellular proliferation which is present in all cell stages except G_0_ (quiescent cells). mRNA levels of *MKI67* were found to be significantly upregulated in healthy fibroblasts by THC (*p* < 0.01) and CBD (*p* < 0.05), as well as by CBD in SIPS fibroblasts (*p* < 0.01) compared to the corresponding vehicles (Figure 8D). Next, we decided to measure levels of proliferating cell nuclear antigen (PCNA), which is a DNA clamp that acts as a processivity factor for DNA polymerase, promotes protein recruitment, and is essential for DNA replication. In healthy and SIPS fibroblasts, no difference in PCNA levels was observed (Figure 9C). After thoroughly looking at pro-proliferative genes and proteins, we decided to study the pro-apoptotic protein Bcl-2 homology 3 interacting-domain death agonist (BID) to see if apoptosis was affected. We found BID was significantly upregulated in SIPS fibroblasts compared to the untreated (*p* < 0.01), and CBD significantly reduced SIPS-induced apoptosis compared to the vehicle (*p* < 0.01, Figure 9D).

As senescence and aging result in replicative senescence, tumor suppressor genes are often dysregulated in senescence phenotypes [35]. Therefore, we looked at p16, p21, and p53 proteins and their associated genes *CDKN1A, CDKN2A,* and *TP53.* qPCR analysis in healthy fibroblasts showed phytocannabinoids generally increased expression of these genes while THC significantly increased levels of *CDKN1A* (*p* < 0.05, Figure 8B) compared to the vehicle, however, Western blot analysis showed no differences between healthy groups for p16 (Figure 9G). However, in SIPS fibroblasts, p16 expression tended to increase, and it was significantly higher after THC treatment (*p* < 0.05, Figure 9G). *CDKN2A* mRNA levels appeared to be increased in healthy fibroblasts exposed to THC (*p* = 0.17) and CBD (*p* = 0.07) compared to the vehicle, while no change was seen in SIPS fibroblasts (Figure 8A). Simultaneously, no changes in p21 protein expression were seen in healthy fibroblasts, but CBD upregulated p21 expression in SIPS fibroblasts compared to the vehicle (*p* < 0.05, Figure 9F). As we have previously shown, p16 and p21 protein levels are not significantly upregulated in SIPS fibroblasts, while the abundance of p53, the principal tumor suppressor protein, increased [17]. In this study, *TP53* mRNA levels were significantly increased in healthy fibroblasts exposed to THC compared to the vehicle (*p* < 0.05, Figure 8C), however, no effect was seen in SIPS fibroblasts. In contrast, uninduced levels of p53 protein trended higher in SIPS fibroblasts, while THC and CBD appeared to downregulate p53 in senescent fibroblasts, although it was not significant (Figure 9E).

Since we saw alterations in p53 and p21 protein levels and *CDKN1A* and *TP53* mRNA levels, we decided to look at the upstream transcription factor c-Jun that is known to regulate these tumor suppressors. In addition, c-Jun is necessary for progression through the G1 phase of the cell cycle via direct transcriptional control of the cyclin D1 gene [36]. We found that c-Jun protein levels were non-significantly upregulated in our SIPS vehicle compared to the healthy vehicle and untreated fibroblasts (*p* = N.S., Figure 9I). Although no significant differences were induced by THC or CBD in SIPS fibroblasts, CBD appeared to lower c-Jun protein levels compared to the vehicle (Figure 9I).

The nuclear factor kappa-light-chain-enhancer of activated B cells (NF-κB) protein regulates the expression of numerous genes associated with cell survival, proliferation, and differentiation. In addition, NF-κB is associated with apoptosis. The mRNA expression of *NF-κB* was not significantly affected by THC or CBD in CCD-106Sk fibroblasts (Figure 8E). In contrast, Western blot analysis showed significant differences between SIPS and healthy fibroblasts (*p* < 0.05, Figure 9H), but THC and CBD did not significantly alter NF-κB protein levels compared to the vehicle (Figure 9I) in either healthy or senescent fibroblasts.

Lastly, we looked at mRNA levels of *growth differentiation factor 11* (*GDF11*). GDF11 is a cytokine that is a member of the transforming growth factor β (TGFβ) family. GDF11 is involved in the regulation of cell growth, proliferation, and differentiation, with numerous roles in physiological organogenesis. In the skin, GDF11 is associated with youthful phenotypes and can inhibit inflammatory responses. The main effect of GDF11 is to promote *COL1A1* and *COL3A1* expression [37]. Therefore, we measured levels of *GDF11* and found CBD boosted mRNA levels in healthy fibroblasts (*p* < 0.05) compared to the vehicle, and THC demonstrated a similar trend (*p* = 0.07, Figure 8F). In SIPS fibroblasts, no significant trend was seen (Figure 8F).

### 3.7. Effects of Phytocannabinoids on Metabolic Regulators and Cannabinoid Receptors

**Figure 10:**
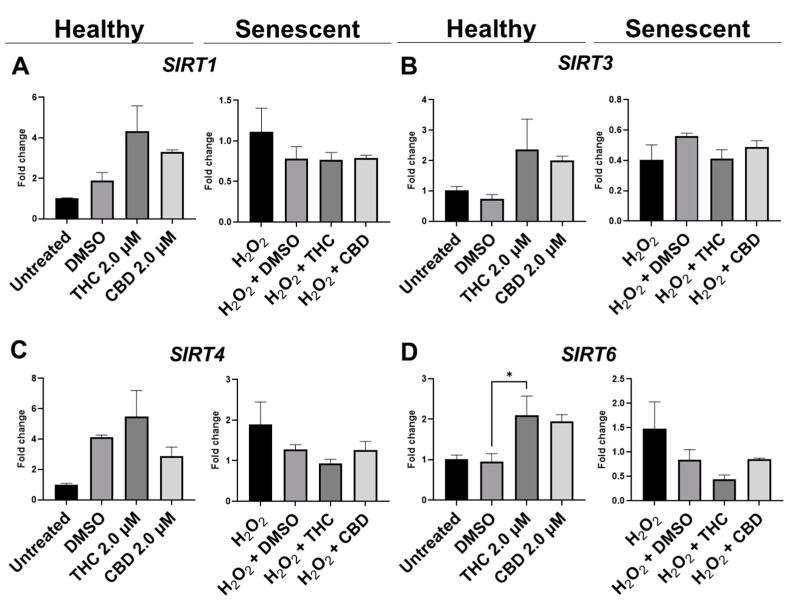
Effects of phytocannabinoids on mRNA levels of metabolic regulators in CCD-1064 human dermal fibroblasts. mRNA levels were presented as a ratio of the target gene relative to *GAPDH* for expression of (**A**) *SIRT1*, (**B**) *SIRT3*, (**C**) *SIRT4*, and (**D**) *SIRT6*. Data were analyzed with an ANOVA test followed by a Dunnett’s post-hoc multiple comparison test compared to the vehicle. Bars represent mean ± SEM, *n* = 3. CBD, cannabidiol; DMSO, dimethyl sulfoxide (vehicle); THC, delta-9-tetrahydrocannabinol. The asterisk shows significant difference (*p* < 0.05).

**Figure 11:**
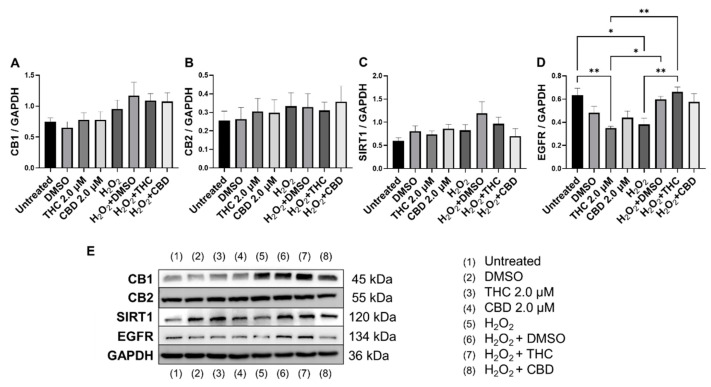
Effects of phytocannabinoids on cannabinoid receptors and metabolic regulators in CCD-1064 human dermal fibroblasts (PDL 24). Relative densitometry was presented as a ratio of the target protein relative to GAPDH for expression of (**A**) CB1, (**B**) CB2, (**C**) SIRT1, and (**D**) EGFR, (**E**) representative images blots with each protein detected. Original membranes can be seen in Figures S21–S25. Data were analyzed with an ANOVA test followed by Tukey’s post-hoc multiple comparison test. Bars represent mean ± SEM, *n* = 3. Significance is indicated using the following scale: * *p* < 0.05, ** *p* < 0.01. CBD, cannabidiol; DMSO, dimethyl sulfoxide (vehicle); THC, delta-9-tetrahydrocannabinol.

To determine the effects of pCBs on changes in metabolic regulation, we tested the expression of sirtuins (SIRT) such as SIRT1 (nuclear), SIRT3 (mitochondrial), SIRT4 (mitochondrial), and SIRT6 (nuclear). The expression of the main “longevity” SIRT1 demonstrated an increasing trend in the THC group (*p* = 0.07) and unaffected in the CBD group of healthy fibroblasts (Figure 10A). Interestingly, Western blot analysis of SIRT1 did not detect any significant alterations in levels of SIRT1 after pCBs application in healthy or SIPS fibroblasts (Figure 11C). Next, we tested other *SIRT*s to see if THC or CBD altered other metabolic regulators. Similarly, THC showed a non-significant trend to increase *SIRT3* (*p* = 0.13, Figure 10B) and *SIRT4* (*p* = 0.61, Figure 10C) expression in healthy fibroblasts compared to the vehicle with no changes seen in SIPS fibroblasts. At the same time, THC significantly increased *SIRT6* mRNA levels in healthy fibroblasts compared to the vehicle (*p* < 0.05), while CBD demonstrated a similar trend (*p* = 0.07, Figure 10D).

Epidermal growth factor receptor (EGFR) is an ErbB receptor tyrosine kinase that is activated by epidermal growth factors. EGFR is involved in a variety of cell signaling pathways that control cell division and survival. EGFR levels have been shown to decrease with age in dermal fibroblasts [38]. Surprisingly, protein levels after THC application in healthy CCD-1064Sk fibroblasts were found to be significantly lower (*p* < 0.05) compared to the vehicle, albeit EGFR levels were significantly lower in SIPS fibroblasts compared to untreated (*p* < 0.05) and were ameliorated by THC in SIPS fibroblasts (*p* < 0.01, Figure 11D).

Lastly, we measured protein levels of the two main canonical cannabinoid receptors, CB1 and CB2, which are known to interact with THC and CBD. As a self-regulatory mechanism, the expression of cannabinoid receptors is altered by receptor activation. Due to this self-regulatory mechanism, alterations in expression can serve as a biomarker for a response to cannabinoids [39]. Surprisingly, we found no significant differences between any groups for both CB1 (Figure 11A) and CB2 (Figure 11B); however, in healthy fibroblasts, a trend to increase in CB1/2 expression was observed in response to pCBs (Figure 11A,B) compared to the vehicle.

Based on these findings, we can assume pCBs mildly preserve ECM components, alter cell cycle regulators, and potentiate metabolic activity in dermal fibroblasts, potentially via a mechanism independent of CB1 and CB2.

## 4. Discussion

Despite numerous studies on cannabis, there is almost no data about the anti-aging or rejuvenation properties of cannabis on the skin. Based on empirical and modern scientific data, cannabis has shown multiple beneficial effects for the treatment of age-related conditions, skin disorders, and delaying senescence [40].

Here, we tested the effects of the major phytocannabinoids found within cannabis, THC, and CBD, on healthy and SIPS fibroblasts. We utilized our previously established SIPS model [17] to study in vitro anti-aging effects and complemented this work with the use of replicative senescent human dermal fibroblasts. We observed a cannabinoid-induced reduction in multiple cellular senescence biomarkers, including β-galactosidase (Figure 3) and cyclin D1 (Figure 9A). In addition, we showed that both THC and CBD have dose-dependent effects on cellular viability, morphology, secretory phenotype, and wound healing. Subsequently, using replicative senescence cells, we showed THC improves wound healing better than common anti-aging nutrient signaling regulators, metformin, rapamycin, and triacetylresveratrol. Data are summarized in Table 1.

The results of the morphological analysis of senescent fibroblasts treated with pCBs demonstrate pCBs are well tolerated, and typical features and architecture of the healthy cells are well preserved. However, at higher concentrations, THC and CBD demonstrated cytotoxic effects. Structural deviations in prematurely aged groups exposed to THC and CBD were associated with slight deformations and increased transparency; in contrast, no significant changes were observed in the pCBs-treated healthy fibroblasts. Merve and colleagues (2022) also reported no changes in cardiomyocyte morphology or viability in response to THC in the low doses (100 ng/mL), whereas treatment with primary THC metabolites 11-hydroxy-D9-THC (THC-OH) and 11-nor-9-carboxy-D9-tetrahydrocannabinol (THC-COOH) in higher doses (250–100 ng/mL) resulted in increased cell death and significant deterioration in cellular architecture [41]. Recent studies have shown that cannabinoids affect the actin cytoskeleton and other components involved in cellular motility, proliferation, and many biological functions in glioblastoma cell lines [42]. Moreover, a cannabinoid-induced reduction in cell growth and a delay in cell division has been observed in the protozoan *Tetrahymena pyriformis* following THC (3.2–24 µM) exposure [43]. This data aligns with our findings that 7.5 µM and higher concentrations of THC and CBD exert cytotoxic effects on healthy dermal fibroblasts.

Analysis of the nuclear architecture showed THC altered nuclear architecture in both healthy and SIPS fibroblasts (Figure 5 and Figure S4). In contrast, CBD applications were almost entirely unchanged in healthy or SIPS fibroblasts, except in the nuclear area and perimeter (Figure 5A and Figure S1). Many changes were seen in THC-treated healthy cells, while some changes were seen in senescent cells treated with THC, while CBD exhibited almost no changes. In SIPS fibroblasts, THC increased eccentricity (Figure 5C) and reduced circularity (Figure 5D). There was a noticeable difference in the number of gigantic or minuscule irregular nuclei (Figure S1).

To elucidate the underlying mechanisms involved in the aging process and identify potential targets for pCBs, the expression of genes involved in cell-cycle regulation, ECM maintenance, and metabolic response were analyzed after pCB exposure. Often, we observed an alteration in mRNA levels induced by THC, specifically in healthy fibroblasts (Figure 6, Figure 8, and Figure 10). This matched our nuclear architecture data as THC often induced nuclear changes in healthy fibroblasts (Figure 5), which would likely lead to changes in transcription.

Although we noticed a mild elevation in *CDKN2A* mRNA levels and significant increases in *CDKN1A* and *TP53* mRNA by THC (Figure 8), minimal differences in the associated proteins were noted. SIPS did not induce any significant changes in p16, p21, or p53 compared to the control (Figure 9). However, THC or CBD did cause slight changes in p16, p21, or p53 protein levels, CBD increased p21 levels in SIPS fibroblasts (Figure 9). p16, p21, and p53 can inhibit cell cycling and induce senescence by interacting with CDKs or CDK inhibitors resulting in inefficient G1-to-S-phase progression. Similarly, CDK2 was unaltered (Figure 9). Senescence activation is not solely dependent on p53, p21, or p16, suggesting separate, independent regulatory networks in the aging process and in this model [44]. Meanwhile, c-Jun is also required for progression through the G1 phase of the cell cycle and directly affects transcriptional control of cyclin D1 [36]. Besides, c-Jun acts as a negative regulator of p53 and p21 expressions and establish a mechanistic link between c-Jun-dependent mitogenic signaling and cell-cycle regulation [45]. Our experiments showed no significant differences in c-Jun protein levels after phytocannabinoid treatment; however, levels appeared to be increased by SIPS, while CBD appeared to decrease c-Jun levels (Figure 9). This apparent increase is in line with increased levels of cyclin D1 in SIPS fibroblasts, which is stereotypical of senescence, and was ameliorated by pCBs exposure (Figure 9). These changes likely explain the increase in proliferative activity of SIPS fibroblasts (Figure 2) due to the restoration of proper cell cycling suggested by amelioration of c-Jun and cyclin D1 protein levels in groups exposed to the THC and CBD treatments.

The main functional activity of fibroblasts is to produce and maintain ECM, such as collagen, elastin, and hyaluronan. With age, their functional capacity declines leading to the appearance of wrinkles, sagging, and dull skin [9,10]. One of the key features of senescent cells is a phenotypic change into a secretory state represented by the release of various inflammatory cytokines, growth factors, enzymes, and ECM proteins known as the senescence-associated secretory phenotype (SASP). To that end, numerous pathways have been shown to regulate SASP: NF-κB, mammalian target of rapamycin (mTOR), p38, mitogen-activated protein kinase (MAPK), sirtuin 3/5 (SIRT3/5), transforming growth factor-beta (TGF-β), H2A histone family member X (γH2AX), ATM induced in response to DNA damage response (DDR), macroH2A1 histone variants, Janus kinases 1/2 (JAK1/2), IL-1α, etc. [46,47,48]. Almost all of these signaling pathways converge at NF-κB, which in turn modulates numerous genes associated with cell survival, proliferation, and differentiation [44,49,50]. We determined moderately upregulated (*p* > 0.05) expression of *NF-κB* in healthy fibroblasts treated with CBD and THC (Figure 8E) that was partially validated by Western blot analysis (Figure 9H). Simultaneously, *NF-κB* appeared to be downregulated in senescent fibroblasts compared to healthy fibroblasts (Figure 8), which was in line with Western blot data, except for CBD treatment of SIPS fibroblasts (Figure 9H). Recent reviews state that activation of NF-κB transcription factor is vital for host defense through innate or adaptive immune system activation. As soon as a harmful agent is eradicated, NF-κB signaling needs to be downregulated to maintain tissue homeostasis required to prevent inflammation, autoimmune disease, and oncogenesis [49,51].

Based on numerous literature and research data, another critical component in cell wellbeing is metabolic regulation, which was altered in senescent fibroblasts. One of the potent metabolic regulators is the family of nicotinamide dinucleotide (NAD+)-dependent protein deacetylases termed sirtuins [47]. Sirtuins are involved in the regulation of DNA repair, glucose output, insulin sensitivity, fatty acid oxidation, fat differentiation, neurogenesis, inflammation, and aging. In our research, we focused on changes in the expression of SIRT1, SIRT3, SIRT4, and SIRT6, which are in mitochondria and nuclei and tightly associated with maintaining cellular homeostasis and reversing some aspects of aging [52]. Thus, SIRT1 is known to deacetylate transcription factors contributing to cellular regulation (reaction to stressors, longevity). Nuclear SIRT1 and SIRT6 regulate cellular responses to energy demands in most tissues through the activity of key transcription factors and cofactors, whereas mitochondrial sirtuins, SIRT3, SIRT4, and SIRT5, control the functioning of mitochondrial enzymes in response to fasting and calorie restriction [53]. It’s worth noting that SIRT4 and SIRT7 repress fatty acids oxidation, insulin secretion, and mitochondrial biogenesis, increasing lipogenesis and liver lipids accumulation. On the contrary, SIRT2, SIRT3, and SIRT6 are positive regulators [54].

We observed no changes in SIRT expression between any groups; however, *SIRT6* mRNA levels were upregulated after THC exposure in healthy fibroblasts (Figure 10D). In cultured cells, embryonic and neonatal tissues, SIRT1 was demonstrated to deacetylate and thereby deactivate the p53 protein, stimulating autophagy by preventing the acetylation of proteins and thereby reducing the risk of age-related diseases [55,56]. Due to this, we decided to look at SIRT1 protein levels in healthy and SIPS fibroblasts. Unfortunately, pCBs were ineffective in both healthy and SIPS groups, with no differences found (Figure 11C).

Previous work shows that SIRT4 is upregulated in various cell lines following senescence and increases with age [57]. Also, SIRT4 transcript levels were decreased in old mouse spermatogonia stem cells after treatment with the lifespan-enhancing drug rapamycin [58]. In normal human epidermal keratinocytes, SIRT4 was expressed inversely to SIRT3, and they both followed a temporal cycle of expression, whereas after UVB and H_2_O_2_ exposure, *SIRT3* and *SIRT4* mRNA levels both increased, and the temporal cycle of expression was lost [59]. In human dermal fibroblasts undergoing replicative or stress-induced senescence triggered by UVB or γ-irradiation, *SIRT4* mRNA and protein levels were also increased [60]. Furthermore, SIRT4 has been shown to prevent the binding of manganese superoxide dismutase, leading to elevated ROS accumulation following UV damage that could contribute to aging-related phenotypes by promoting increased ROS generation [61]. In our model, *SIRT3* and *SIRT4* mRNA levels appeared to be increased by THC but were not significant (*p* > 0.05, Figure 10B,C).

As a first step to study the potential role of phytocannabinoids in the rejuvenation properties of collagen restoration, elastin, and hyaluronic acid production in senescent fibroblasts, we tested mRNA transcripts and protein levels of main ECM components.

In healthy fibroblasts, THC upregulated *COL1A1* expression (Figure 6A). However, COL1A1 protein level did not increase in response to THC (Figure 7A) but trended higher after CBD exposure (*p* > 0.05, Figure 7A). Simultaneously, CBD showed no effect on *COL1A1* mRNA or protein (Figure 6 and Figure 7). Additionally, we found *ELN* mRNA to be upregulated after THC (*p* < 0.001) and CBD (*p* < 0.05) exposure in healthy fibroblasts (Figure 6C) but no effects in SIPS fibroblasts. Conversely, ELN protein levels were unchanged in healthy fibroblasts, downregulated in SIPS fibroblasts (*p* < 0.01), and upregulated by CBD exposure (*p* < 0.05, Figure 7C) compared to the controls. As expected, collagen expression in prematurely aged dermal fibroblasts compared to healthy ones was reduced regardless of treatment (Figure 7A). Despite this, it was noted that CBD tended to increase COL1A1 expression levels. These findings agree with the available literature [62], confirming the downregulation of COL1A1, COL3A1, ELN, and TIMP1 in senescent fibroblasts.

Interestingly, human dermal fibroblasts from elderly donors were different from the cellular replicative senescence model in the expression of only three genes: LMNA, TIMP1, and TIMP2 [62]. Hence, other aging study models might reveal variations in gene expression that explain discrepancies in the observed results. In our model, MMP2, responsible for basement membrane collagens breakdown and degradation of denatured structural collagens, was minimally altered in response to pCBs treatment in healthy and slightly elevated in SIPS fibroblasts (Figure 6F and Figure 7D). This would suggest that the beneficial effects of pCBs are likely due to an increase in the expression of mRNA and proteins that build the collagen-elastin net of the ECM, which supports the dermis and is not related to the degradation of the ECM environment.

Next, we compared the pCBs effect on wound healing. Interestingly, after 24 h of exposure in the H_2_O_2_-induced senescent group, visual wound enlargement in CCD-1064Sk cell lines was found (Figure 4B). This might be related to the diminished adhesive protein vinculin levels (*p* < 0.01, Figure 7E) and explained by increased cellular detachment resulting in levered adhesive forces potentiated by injury (Figure 2). Although neither THC nor CBD upregulated vinculin, which is decreased in our SIPS model [17], to promote fibroblast migration and skin repair, THC and CBD stimulated fibroblasts’ ability to close the damaged wound, which is in line with upregulation of the proliferative biomarkers (i.e., MKI67) in both healthy and senescent cells. Moreover, a pro-apoptotic BID protein decreased after CBD exposure in SIPS fibroblasts (*p* > 0.01, Figure 9D). Interestingly, alterations in mRNA levels did not always match changes in protein levels. It is possible that the time we chose to analyze the samples captures changes in the protein levels, while changes in mRNA levels may have been quicker and, thus, transient in nature.

Recently, THC was reported to promote wound healing by inducing periodontal fibroblast cell adhesion and migration in a CB2-dependent manner via the activation of focal adhesion kinase and its modulation of MAPK activities [63]. The authors showed that the effect of cannabinoids on periodontal fibroblast cell adhesion and migration was mainly dependent on CB2. However, the results of our investigation showed that neither CB1 nor CB2 expression was altered in healthy and senescent fibroblasts after pCBs treatment (Figure 11A,B).

Thus, the data gathered here are not sufficient to definitively answer if cannabinoids can completely reverse or stop aging; however, THC is likely useful as a therapeutic to reduce senescent-associated changes (e.g., structural prevention changes of nuclei and scaffolding proteins of ECM such as collagen and elastin, regulation of cell cycle, and conserving cellular functions), while CBD shows modest beneficial effects. In particular, THC shows promise in treating healthy skin to prevent typical aging by boosting ECM production, preserving cell-cycle regulators, and improving wound healing.

## 5. Conclusions

The key findings of our study are:

(i) pCBs have a therapeutic range of 0.5–2 µM in dermal fibroblasts,
(ii) THC and CBD stimulated fibroblasts’ ability to close damaged wounds, while THC induced wound healing better than common nutrient signaling regulators,
(iii) THC reduced morphological alterations in skin cells, potentiated cellular viability via preserving cell-cycle regulators, and boosted ECM production,
(iv) CBD provided few beneficial effects on dermal fibroblasts.
(v) Our data helps fill the gap regarding the potential role of THC/CBD in anti-aging research and demonstrates THC has therapeutic potential for cosmetic applications.

## Figures and Tables

**Table 1:** Summary of the investigated characteristics of healthy and prematurely aged skin fibroblasts after phytocannabinoid treatment.

Characteristic	Young Fibroblasts		Senescent Fibroblasts	
	THC	CBD	H_2_O_2_ + THC	H_2_O_2_ + CBD
Shape and size of the cell	Elongated, small	Elongated, small	Enlarged, flattened	Enlarged, flattened
Nuclear Area	↓	↔	↔	↔
Nuclear Perimeter	↓	↔	↔	↔
Nuclear Eccentricity	↑	↔	↔	↔
Nuclear circularity (roundness)	↓	↔	↔	↔
β-Gal level	↑	↔	↓	↓
BID	↔	↔↑	↔↓	↓
CB1	↔↑	↔↑	↔	↔
CB2	↔↑	↔↑	↔	↔↑
CDK2	↔↑	↔↑	↔↓	↔↓
c-Jun	↔↓	↔	↔	↔↓
Collagen (type I)	↔	↔↓	↔↑	↔↑
Collagen (type III)	↔	↔	↔	↔
Cyclin D1	↔	↔↓	↓	↓
EGFR	↓	↔	↑	↔
Elastin	↔	↔	↔↓	↓
GDF11	↔↑	↑	↔	↔↑
Hyaluronan synthase	↑	↑	↔↑	↔↑
MMP 1	↑	↔	↓	↔↓
MMP 2	↔	↔	↔↑	↔↑
MKI67	↑	↑	↔	↑
NF-κB	↔↑	↔↑	↔↓	↔↑
P16	↔	↔↑	↑	↔↑
P21	↔↓	↔↓	↔	↑
P53	↔↑	↔↑	↔	↔↓
PCNA	↔	↔	↔	↔
SIRT 1	↔↑	↔↑	↔	↔
SIRT 3, 4	↔↑	↔↑	↔↓	↔
SIRT 6	↑	↔↑	↔↓	↔
TIMP 1	↔↑	↔↑	↔↓	↔↓
Vinculin	↔↓	↓	↔	↔
Cell viability	↑	↔	↑	↑
Wound healing	↑	↑	↑	↑

Results compared to vehicle control, DMSO in healthy fibroblasts group, and H_2_O_2_ + DMSO. In senescent fibroblast group. **↓**, Decreased/low; **↑**, Increased/high; **↔**, no change detected; ↔↓, tendency to decrease; ↔↑, tendency to increase.

## Data Availability

Not applicable.

## Supplementary Materials

The following supporting information can be downloaded at: https://www.mdpi.com/article/10.3390/cells11233939/s1, Table S1: Antibodies used for western blots; Table S2: Primer sequences for qPCR analysis; Figure S1: Human skin fibroblasts (CCD-1064Sk), 24 PDL exposed to different concentrations of THC and CBD; Figure S2: Human skin fibroblasts (CCD-1064Sk), 24 PDL after 24 h phytocannabinoids treatment; Figure S3: Comparison of phytocannabinoids with common anti-aging nutrient signaling receptors (NSRs) on wound healing in replicative senescence CCD-1135Sk (PDL 40) human dermal fibroblasts; Figure S4: DAPI-stained Human skin fibroblasts (CCD-1064Sk), 24 PDL exposed to THC and CBD at 2 µM; Figure S5: Original Western blots of CCD-1064k (PDL 24) proteins showing COL1A1 (molecular weight is 140 kDa); Figure S6: Original Western blots of CCD-1064k (PDL 24) proteins showing COL3A1 (molecular weight is 110 kDa); Figure S7: Original Western blots of CCD-1064k (PDL 24) proteins showing Elastin (molecular weight is 70 kDa); Figure S8: Original Western blots of CCD-1064k (PDL 24) proteins showing MMP2 (molecular weight is 72 kDa); Figure S9: Original Western blots of CCD-1064k (PDL 24) proteins showing Vinculin (molecular weight is 124 kDa); Figure S10: Original Western blots of CCD-1064k (PDL 24) proteins showing GAPDH (molecular weight is 36 kDa); Figure S11: Original Western blots of CCD-1064k (PDL 24) proteins showing Cyclin D1 (molecular weight is 34 kDa); Figure S12: Original Western blots of CCD-1064k (PDL 24) proteins showing CDK2 (molecular weight is 33 kDa); Figure S13: Original Western blots of CCD-1064k (PDL 24) proteins showing PCNA (molecular weight is 36 kDa); Figure S14: Original Western blots of CCD-1064k (PDL 24) proteins showing BID (molecular weight is 22 kDa); Figure S15: Original Western blots of CCD-1064k (PDL 24) proteins showing p53 (molecular weight is 53 kDa); Figure S16: Original Western blots of CCD-1064k (PDL 24) proteins showing p21 (molecular weight is 21 kDa); Figure S17: Original Western blots of CCD-1064k (PDL 24) proteins showing p16 (molecular weight is 16 kDa); Figure S18: Original Western blots of CCD-1064k (PDL 24) proteins showing NF-κB (molecular weight is 65 kDa); Figure S19: Original Western blots of CCD-1064k (PDL 24) proteins showing c-Jun (molecular weight is 39 kDa); Figure S20: Original Western blots of CCD-1064k (PDL 24) proteins showing GAPDH (molecular weight is 36 kDa); Figure S21: Original Western blots of CCD-1064k (PDL 24) proteins showing CB1 (molecular weight is 45 kDa); Figure S22: Original Western blots of CCD-1064k (PDL 24) proteins showing CB2 (molecular weight is 55 kDa); Figure S23: Original Western blots of CCD-1064k (PDL 24) proteins showing SIRT1 (molecular weight is 120 kDa); Figure S24: Original Western blots of CCD-1064k (PDL 24) proteins showing EGFR (molecular weight is 134 kDa); Figure S25: Original Western blots of CCD-1064k (PDL 24) proteins showing GAPDH (molecular weight is 36 kDa).
