# Supplementary material for: Phytocannabinoids Stimulate Rejuvenation and Prevent Cellular Senescence in Human Dermal Fibroblasts
Source: Cells. 2022 Dec 6;11(23):3939. doi: 10.3390/cells11233939 (PMC9738082; doi:10.3390/cells11233939)
Supplement: Supplementary file 1 [file cells-11-03939-s001.zip › cells-2037212-supplementary.pdf]

**Table S1.** Antibodies used for western blots

| Antibody | Supplier, Cat No | Dilution |
|---|---|---|
| Mouse anti-NFκB p65 | Santa Cruz, sc-8008 | 1:500 in 5% milk (PBST) |
| Mouse anti-CB1/Cannabinoid Receptor 1/CNR1 (2F9) | Santa Cruz, sc-293419 | 1:100 in 5% milk (PBST) |
| Mouse anti-CB2/Cannabinoid Receptor 2/CNR2 (3C7) | Santa Cruz, sc-293188 | 1:200 in 5% milk (PBST) |
| Mouse anti-COL1A (COL-1) | Santa Cruz, sc-59772 | 1:100 in 5% milk (PBST) |
| Mouse anti-COL3A1 (B-10) | Santa Cruz, sc-271249 | 1:100 in 5% milk (PBST) |
| Mouse anti-elastin (BA-4) | Santa Cruz, sc-58756 | 1:200 in 5% milk (PBST) |
| Mouse anti-SIRT1 (B-7) | Santa Cruz, sc-74465 | 1:100 in 5% milk (PBST) |
| Rabbit p16 INK4A (D7C1M) | Cell Signalling, #80772 | 1:500 in 5% milk (PBST) |
| Rabbit p21 Waf1/Cip1 (12D1) | Cell Signalling, #2947 | 1:500 in 5% milk (PBST) |
| Rabbit MMP-2 (D4M2N) | Cell Signalling, #40994 | 1:500 in 5% milk (PBST) |
| Mouse anti-cyclin D1 (DCS-6) | Santa Cruz, sc-20044 | 1:500 in 5% milk (PBST) |
| Rabbit CDK2 (78B2) | Cell Signalling, #2546 | 1:500 in 5% milk (PBST) |
| Rabbit-c-Jun (60A8) | Cell Signalling, #9165 | 1:200 in 5% milk (PBST) |
| Mouse anti-BID (E-7) | Santa Cruz, sc-514622 | 1:200 in 5% milk (PBST) |
| Mouse anti-EGFR (A-10) | Santa Cruz, sc-373746 | 1:200 in 5% milk (PBST) |
| Mouse anti-vinculin (7F9) | Santa Cruz, sc-73614 | 1:500 in 5% milk (PBST) |
| Mouse anti-p53 (DO-1) | Santa Cruz, sc-126 | 1:500 in 5% milk (PBST) |
| Mouse anti-GAPDH (0411) | Santa Cruz, sc-47724 | 1:1000 in 5% milk (PBST) |
| Bovine anti-Mouse | Santa Cruz, sc-2371 | 1:10000 in 5% milk (PBST) |
| Donkey anti-Rabbit | Santa Cruz, sc-2313 | 1:10000 in 5% milk (PBST) |

**PBST**, 1x Phosphate-Buffered Saline, 0.1 % Tween® 20; **Santa Cruz**, Santa Cruz Biotechnology, Inc., Texas, United States; **Cell Signaling**, Cell Signaling Technologies, Massachusetts, United States

**Table S2.** Primer sequences for qPCR analysis

| Target Gene | Sequence Forward (5' → 3') | Sequence Reverse (5' → 3') |
|---|---|---|
| ***COL1A1*** | CCACGACAAAGCAGAAACATC | GCAACACAGTTACACAAGGAAC |
| ***COL3A1*** | CTGGCATTCCTTCGACTTCT | AGCTTCAGGGCCTTCTTTAC |
| ***GDF11*** | TCTCAGAGCTAGTGTGGTAGAA | CCTCCCGGATCACTTTCAATAG |
| ***ELN*** | CTCAAAGCTGGATTCGCTCTA | AAGGGCAAGGTGGCTATTC |
| ***MMP1*** | CAGAAAGAGACAGGAGACATGAG | GAAGAGTTATCCCTTGCCTATCC |
| ***MMP2*** | AGAGAACCTCAGGGAGAGTAAG | CCTCGAACAGATGCCACAATA |
| ***HAS1*** | GTCTCCAGGGAGGGTATTTATTG | TCCTGATCACACAGTAGAAATGG |
| ***CDKN2A (P16)*** | AGCTGTCGACTTCATGACAAG | GAGCTTTGGTTCTGCCATTTG |
| ***EGFR*** | CAAGGAAGCCAAGCCAAATG | CCGTGGTCATGCTCCAATAA |
| ***MKI67*** | GGAGCCAGGTGACATCATAAA | CATGGATGACGCTGTGAGAA |
| ***CDKN1A (P21)*** | CCTTCCAGCTCCTGTAACATAC | TCGAGAGGTTTACAGTCTAGGT |
| ***SIRT1*** | AGAACCCATGGAGGATGAAAG | TCATCTCCATCAGTCCCAAATC |
| ***SIRT3*** | CCTCCTTCCTAGCATCACATTAC | CCTGGGAGTCACTGTCATTAAA |
| ***SIRT4*** | GAACCTGGAACAGGGACTTT | CTTTGTCAGTGCACCCTACT |
| ***SIRT6*** | CCTCTGACTTGCTGTGTTGT | GAGGGAGTTCACTCCTGTTTAAG |
| ***TP53*** | AGGGATGTTTGGGAGATGTAAG | CCTGGTTAGTACGGTGAAGTG |
| ***NFKB1*** | GAGACATCCTTCCGCAAACT | GGTCCTTCCTGCCCATAATC |
| ***TIMP1*** | TCCCAGATAGCCTGAATCCT | TGCTGGGTGGTAACTCTTTATT |
| ***GAPDH*** | CAGGAGGCATTGCTGATGAT | GAAGGCTGGGGCTCATTT |

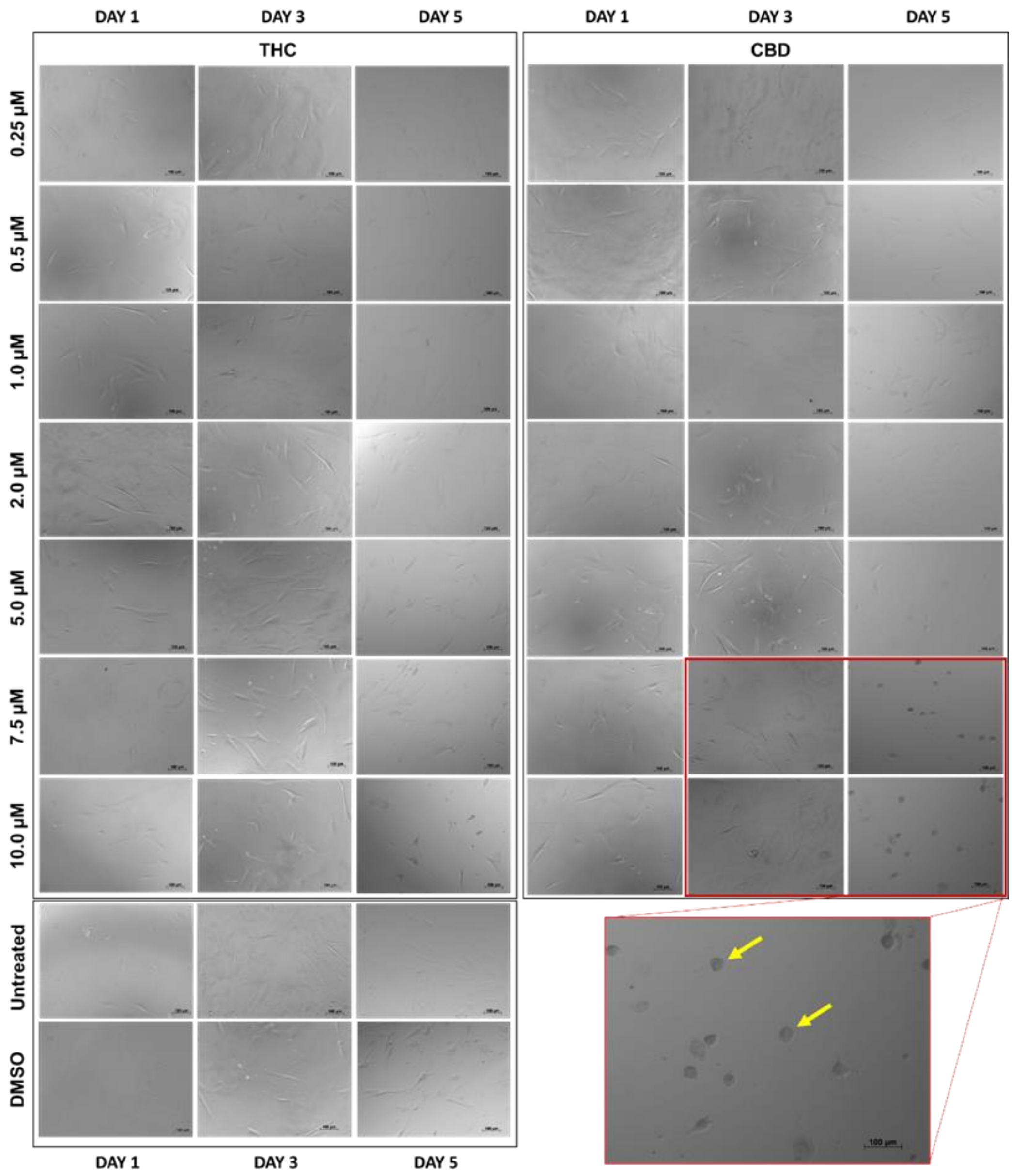


***Figure S1***. Human skin fibroblasts (CCD-1064Sk), 24 PDL exposed to different concentrations of THC and CBD. The figure represents gradual changes in cell quality and quantity on the 1st, 3rd, and 5th day of treatment. Arrows depict changes in shapes – coin-like round fibroblasts. DMSO, dimethyl sulfoxide (vehicle).

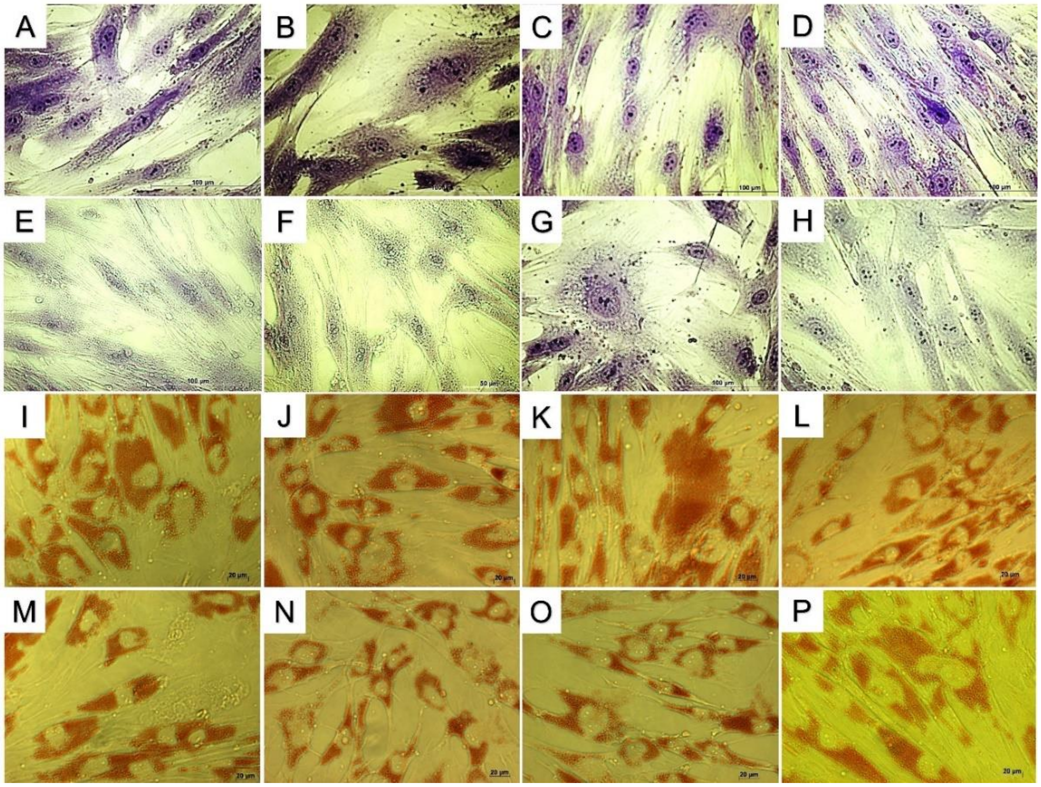

***Figure S2.*** Human skin fibroblasts (CCD-1064Sk), 24 PDL after 24 hours phytocannabinoids treatment. (**A-H**), Crystal violet staining; (**I-P**), Neutral red staining. (**A,I**) represents untreated fibroblasts; (**B,J**) vehicle (DMSO); (**C,K**) THC 2.0 μM treatment; (**D,H**) CBD, 2.0 μM treatment; (**E,M**), 25 μM $H_2O_2$; (**F,N**) $H_2O_2$+DMSO; (**G,O**) $H_2O_2$+THC; (**H,P**) $H_2O_2$+CBD.

Neutral red staining demonstrates the ability of viable cells to incorporate and bind neutral red dye in the lysosomes (doi:10.1038/nprot.2008.75). We added 100 μL of Neutral red (N7005, Sigma-Aldrich, Saint Louis, USA) dissolved in a cell culture medium (40 μg mL−1) to fibroblasts in each well in a 24-well plate, followed by 4 hours incubation and afterward double washing with 150 μL of PBS. Stained dermal fibroblasts were photographed using a Zeiss Ob-server Z1 epifluorescence microscope with AxioVision Rel 4.8 software.

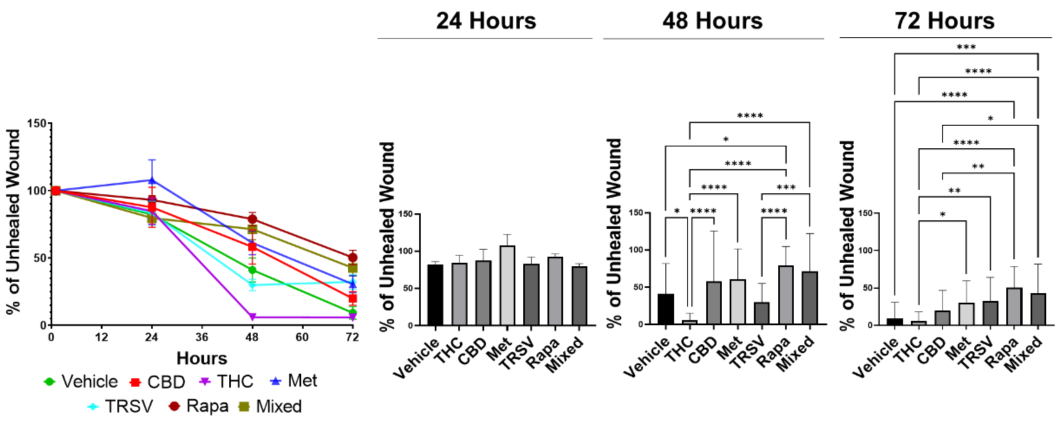


***Figure S3.*** Comparison of phytocannabinoids with common anti-aging nutrient signaling receptors (NSRs) on wound healing in replicative senescence CCD-1135Sk (PDL 40) human dermal fibroblasts. (A) Percentage of unhealed wound was measured at 24, 48 and 72 hours after scratch assay was performed with replicative senescent dermal fibroblasts exposed to THC (2 μM), CBD (2 μM), Met (500 μM), TRSV (10 μM), Rapa (5 μM), or a mixed treatment of all NSRs and pCBs. (B) Percentage of unhealed wound at 24, 48 and 72 hours after scratch assay was performed with senescent dermal fibroblasts exposed to 2 μM of THC or CBD. Data were analyzed with an ANOVA test followed by a Tukey's post-hoc multiple comparison test. Bars represent mean ± SEM, n=6. Significance is indicated using the following scale: *P<0.05, **P<0.01, ***P<0.001, ****P<0.0001. CBD, cannabidiol; DMSO, dimethyl sulfoxide (vehicle); Met, metformin; pCBs, phytocannabinoids; Rapa, rapamycin; THC, delta-9-tetrahydrocannabinol; TRSV, triacetylresveratrol.

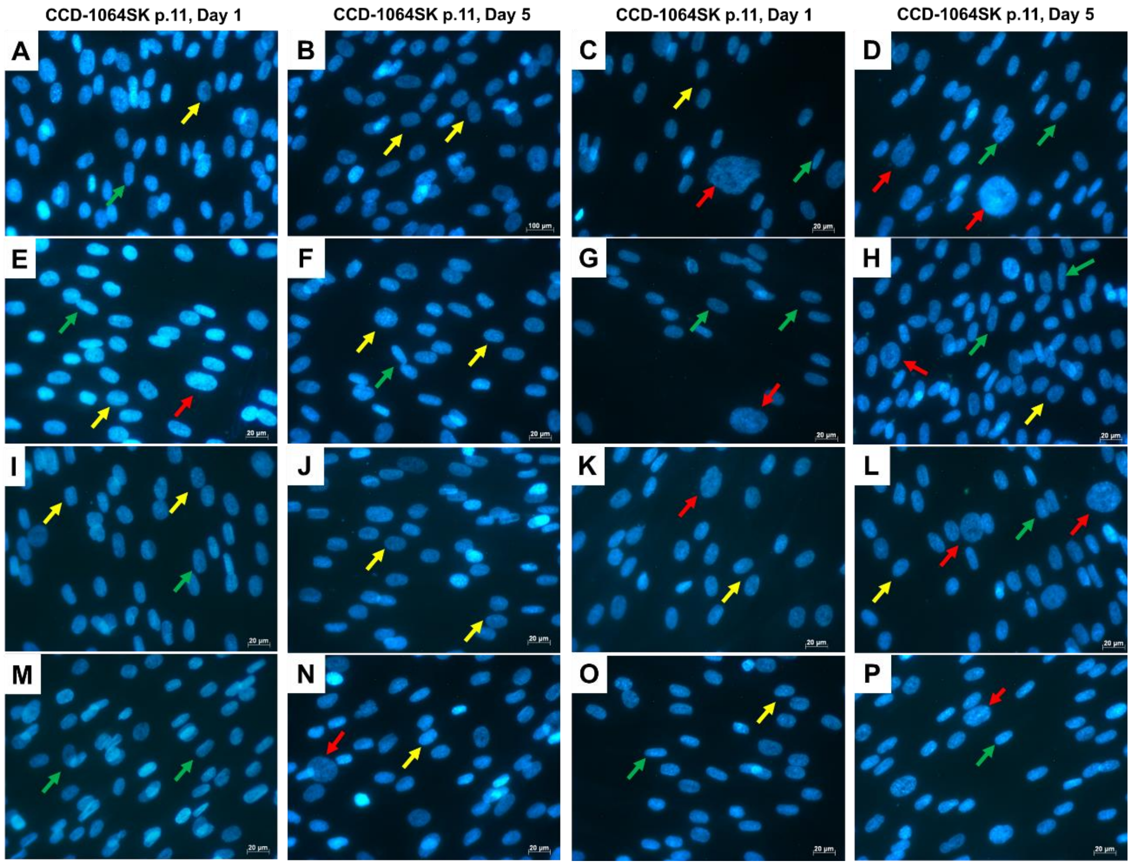


***Figure S4.*** DAPI-stained Human skin fibroblasts (CCD-1064Sk), 24 PDL exposed to THC and CBD at 2 μM. Pictures (**A-P**) represent nuclear changes observed by immunofluorescence microscopy in healthy and senescent fibroblasts treated with cannabinoids on days 1 and 5. Arrow depict changes in nuclear shapes: yellow – round, green – elongated, and red – gigantic/irregular. (**A,B**) Untreated; (**C,D**), 25 μM $H_2O_2$; (**E,F**) DMSO (vehicle); (**G,H**) 25 μM $H_2O_2$+DMSO; (**I,J**) THC; (**K,L**) 25 μM $H_2O_2$+THC; (**M,N**) CBD; (**O,P**) 25 μM $H_2O_2$+CBD.

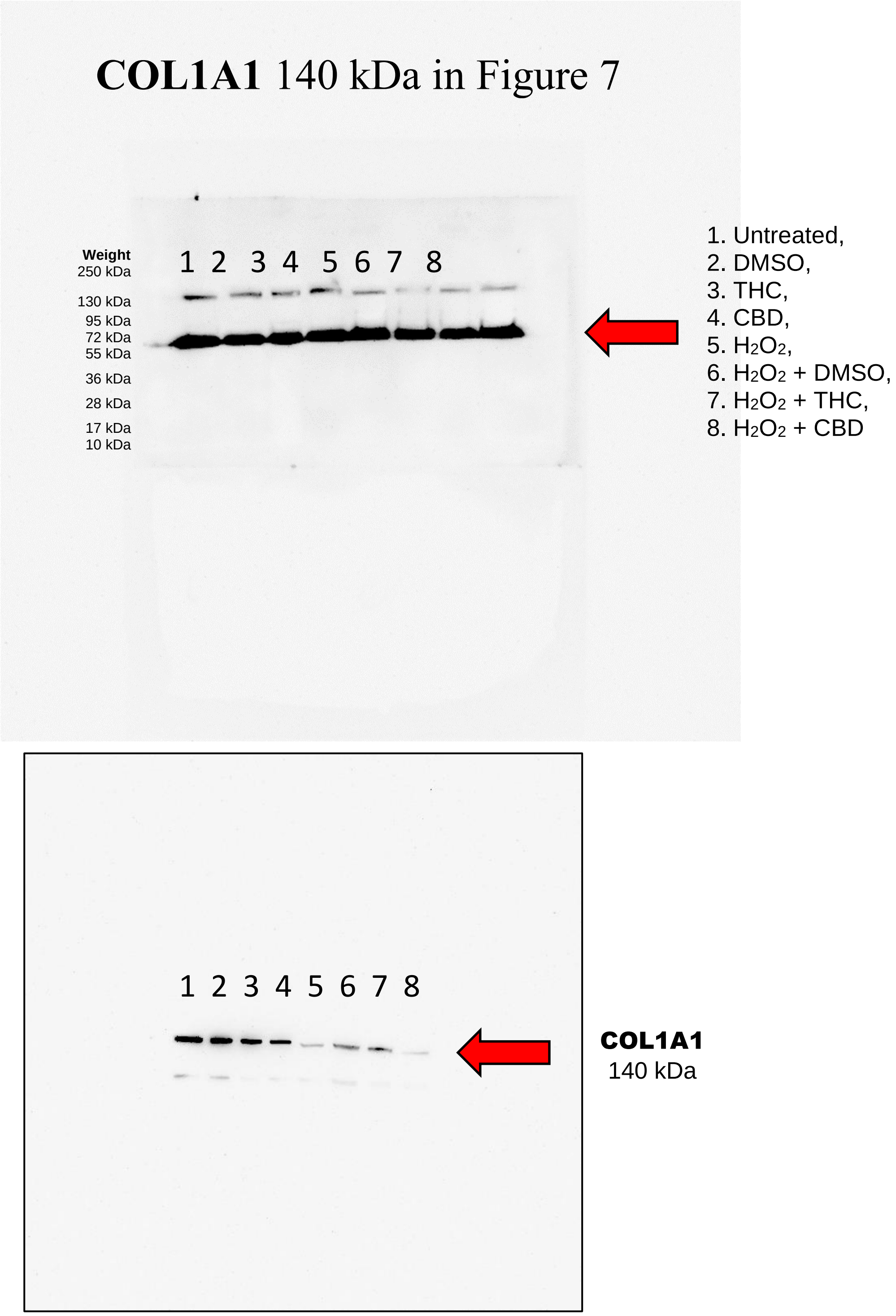


***Figure S5.*** Original Western blots of CCD-1064k (PDL 24) proteins showing COL1A1 (molecular weight is 140 kDa). Red arrow indicates COL1A1 bands. Bands in the first blot was used in Figure 7. These blots have been stripped and re-probed.

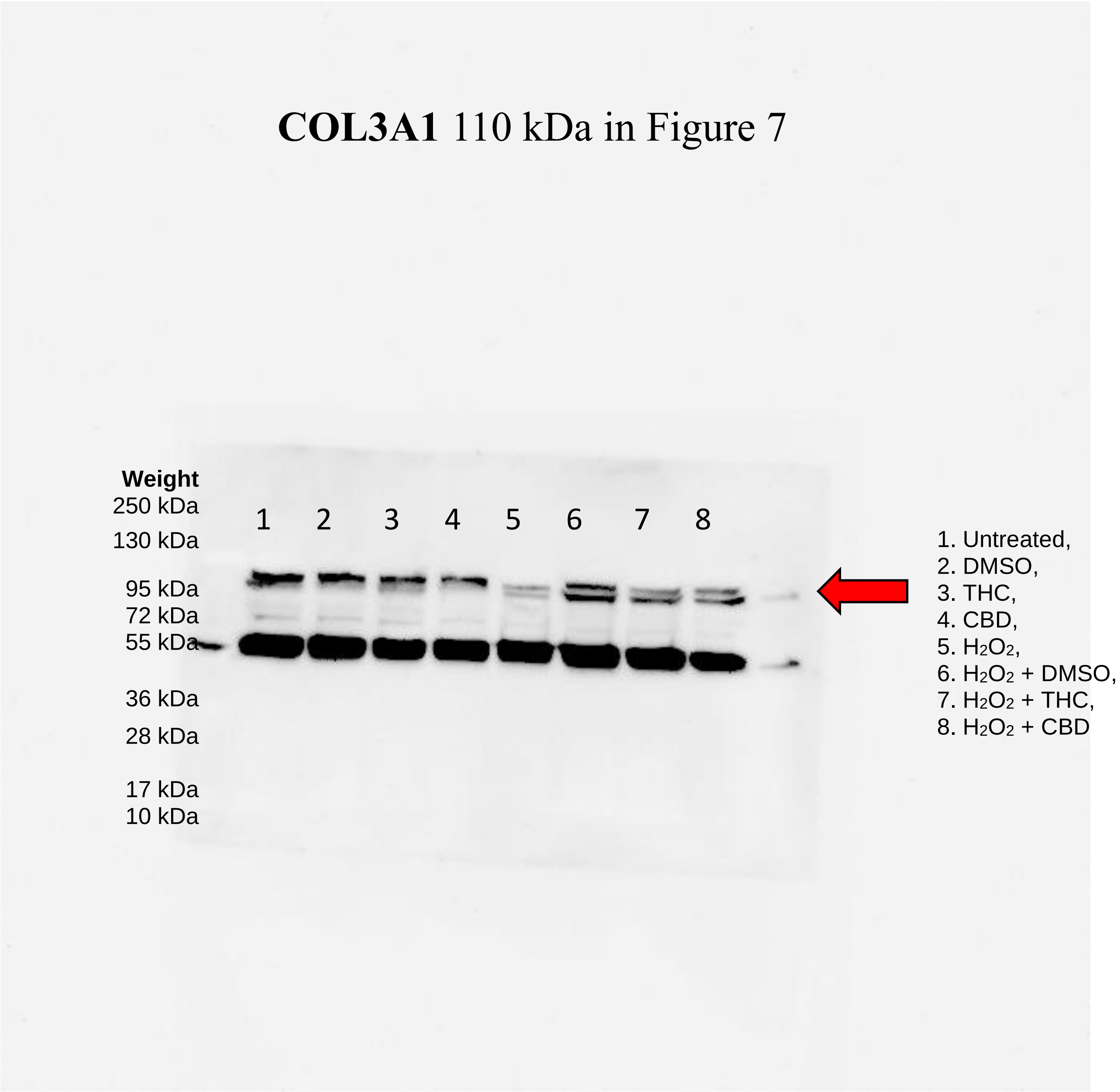


***Figure S6.*** Original Western blots of CCD-1064k (PDL 24) proteins showing COL3A1 (molecular weight is 110 kDa). Red arrow indicates bands shown in Figure 7. This blot has been stripped and re-probed.

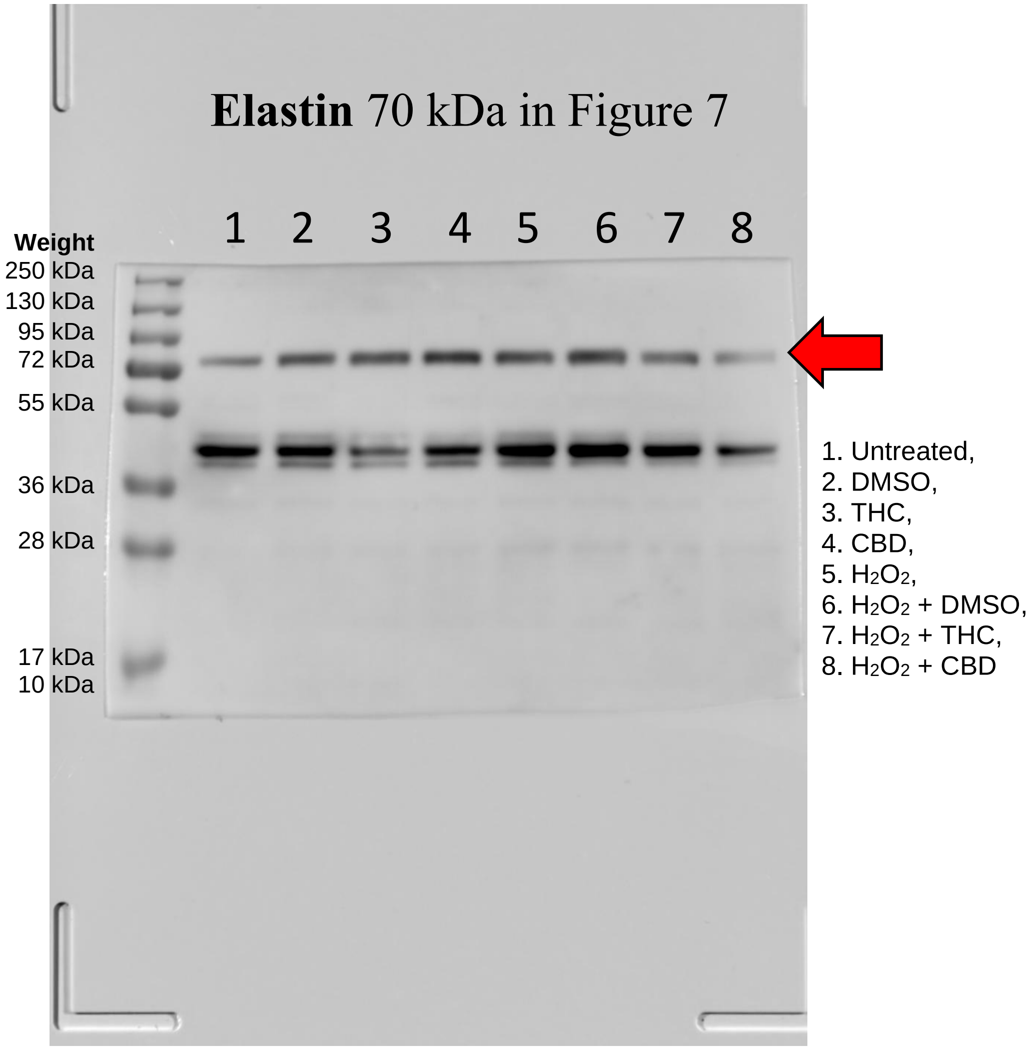


***Figure S7.*** Original Western blots of CCD-1064k (PDL 24) proteins showing Elastin (molecular weight is 70 kDa) Bands shown in Figure 7. This blot has been stripped and re-probed.

**MMP2** 72 kDa in Figure 7

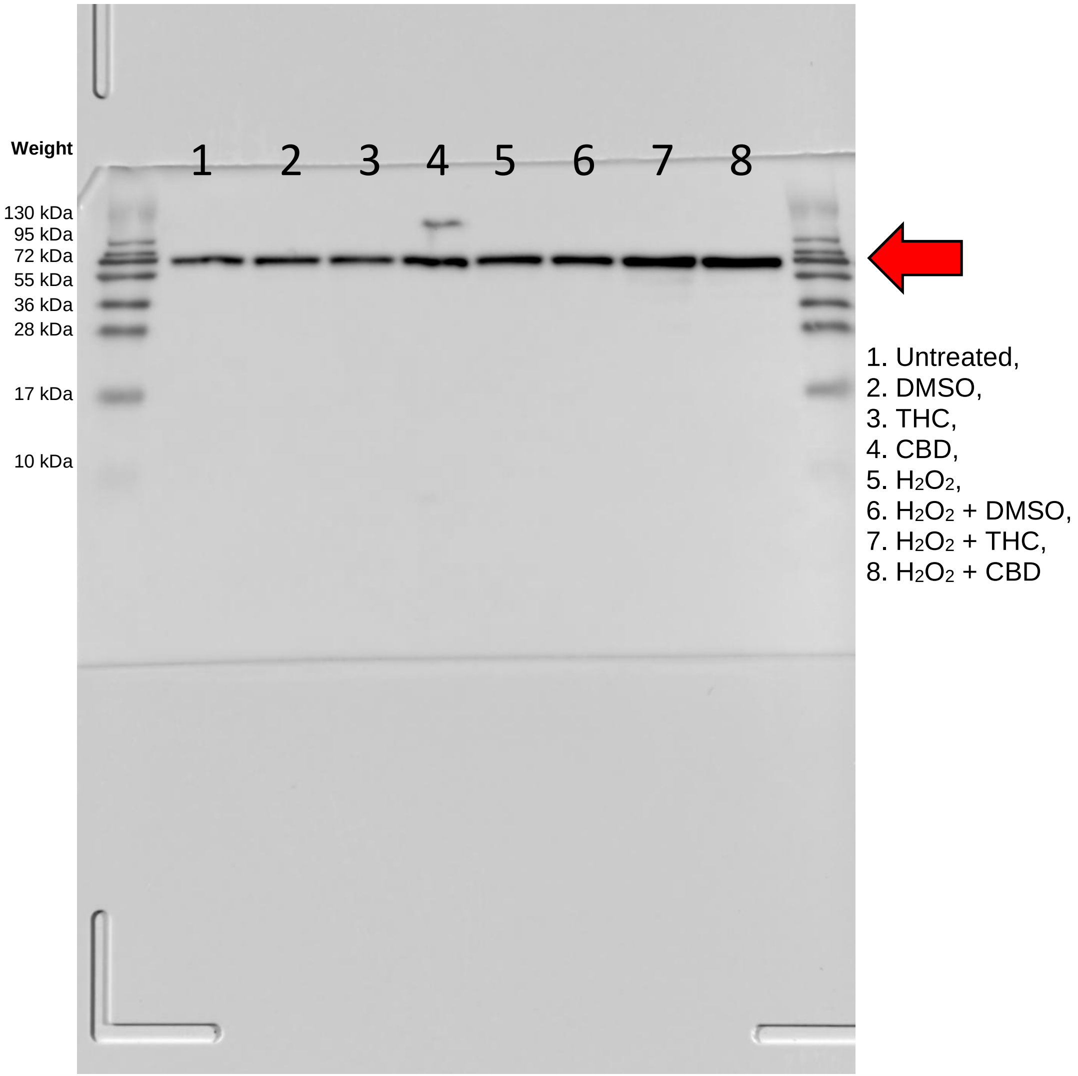


***Figure S8.*** Original Western blots of CCD-1064k (PDL 24) proteins showing MMP2 (molecular weight is 72 kDa) Bands shown in Figure 7.

**Vinculin** 124 kDa in Figure 7

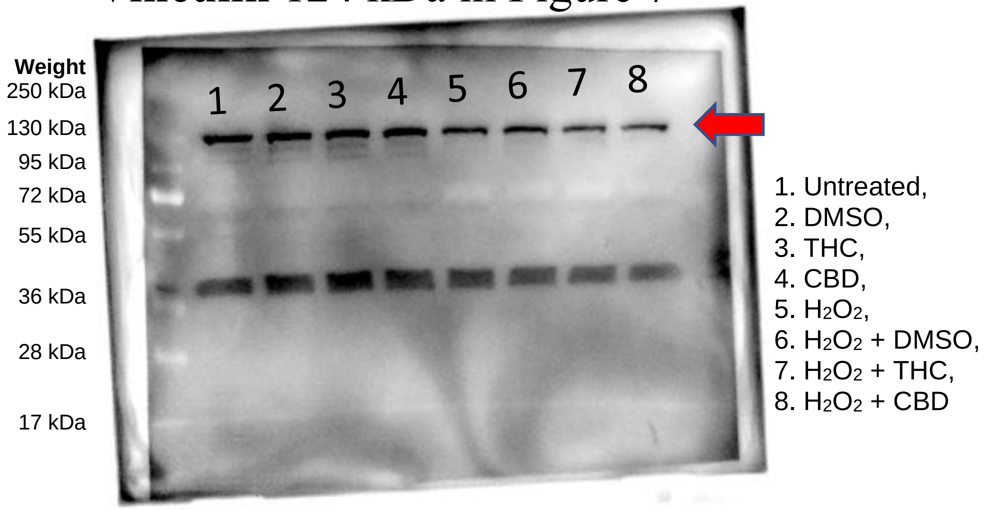


***Figure S9.*** Original Western blots of CCD-1064k (PDL 24) proteins showing Vinculin (molecular weight is 124 kDa). Red arrow indicates bands shown in Figure 7. This blot has been stripped and re-probed.

## **GAPDH** 36 kDa in Figure 7

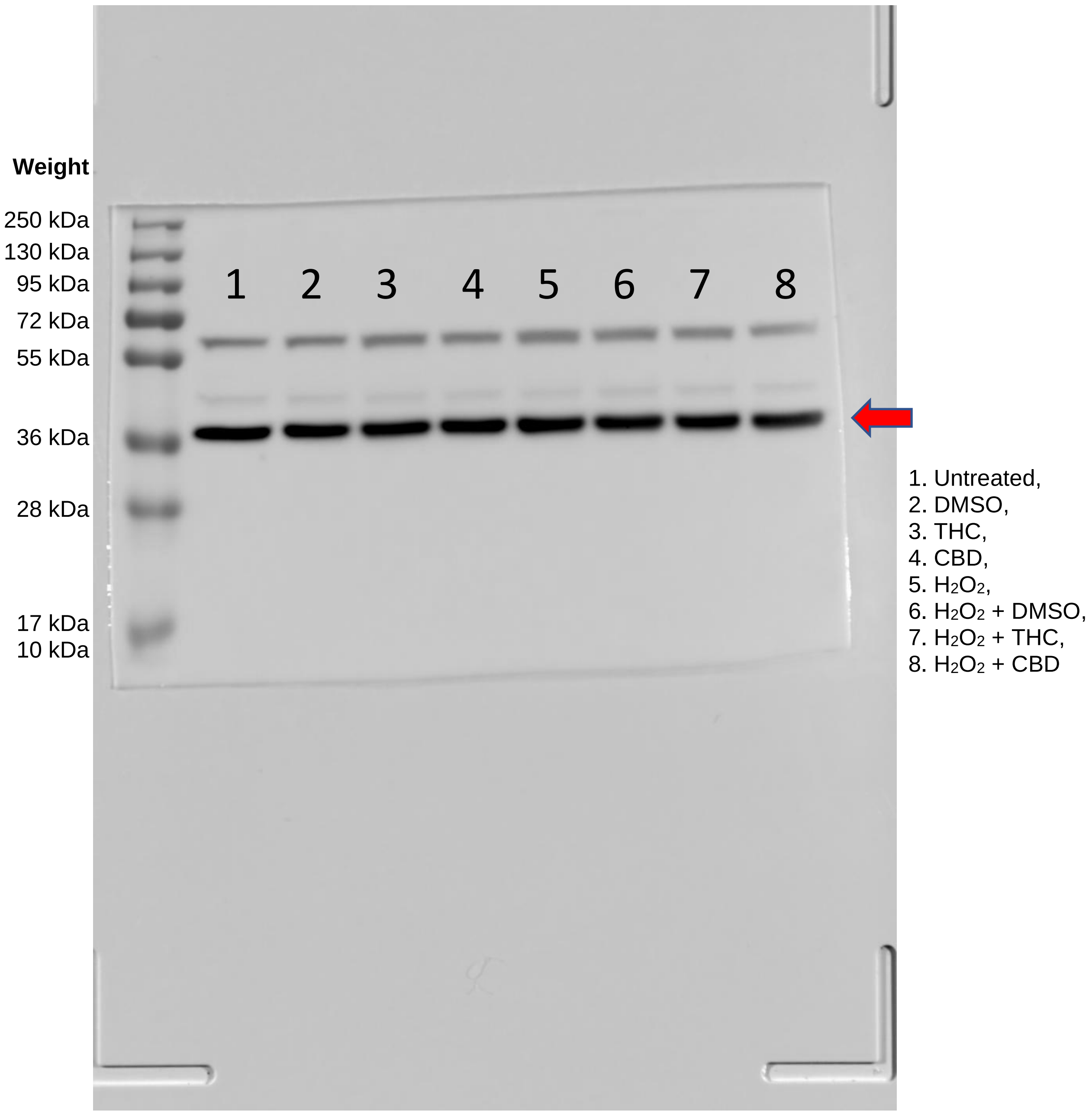


***Figure S10.*** Original Western blots of CCD-1064k (PDL 24) proteins showing GAPDH (molecular weight is 36 kDa). Bands shown in Figure 7. This blot has been stripped and re-probed.

**Cyclin D1** 34 kDa in Figure 9

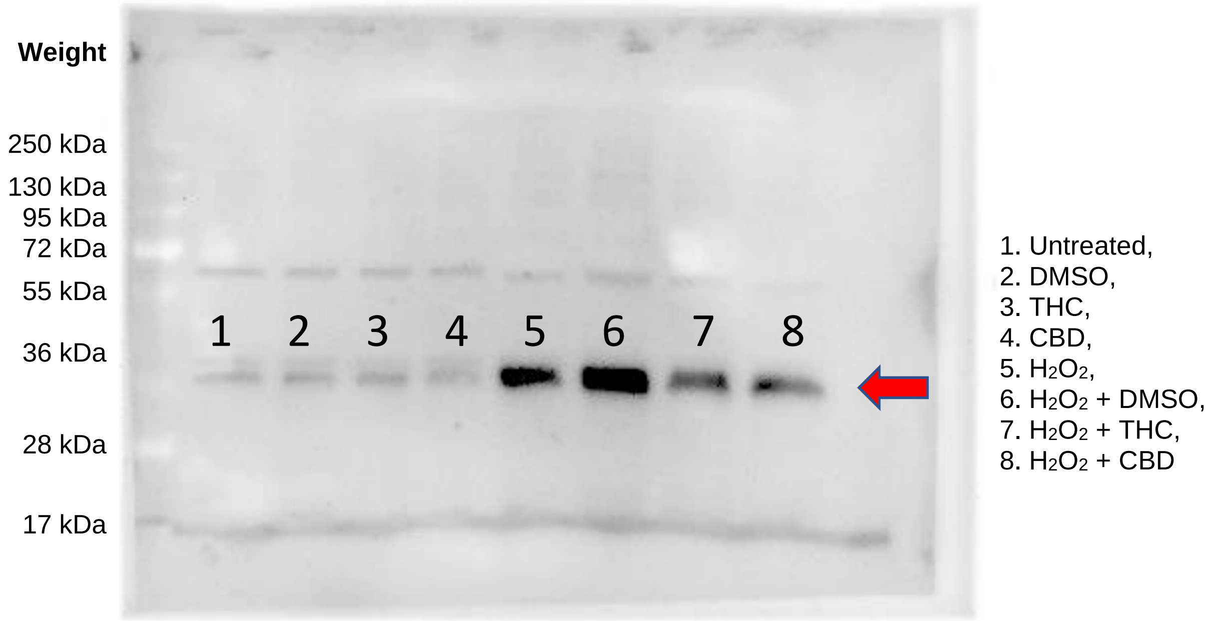


***Figure S11.*** Original Western blots of CCD-1064k (PDL 24) proteins showing Cyclin D1 (molecular weight is 34 kDa) Red arrow indicates bands shown in Figure 9. This blot has been stripped and re-probed.

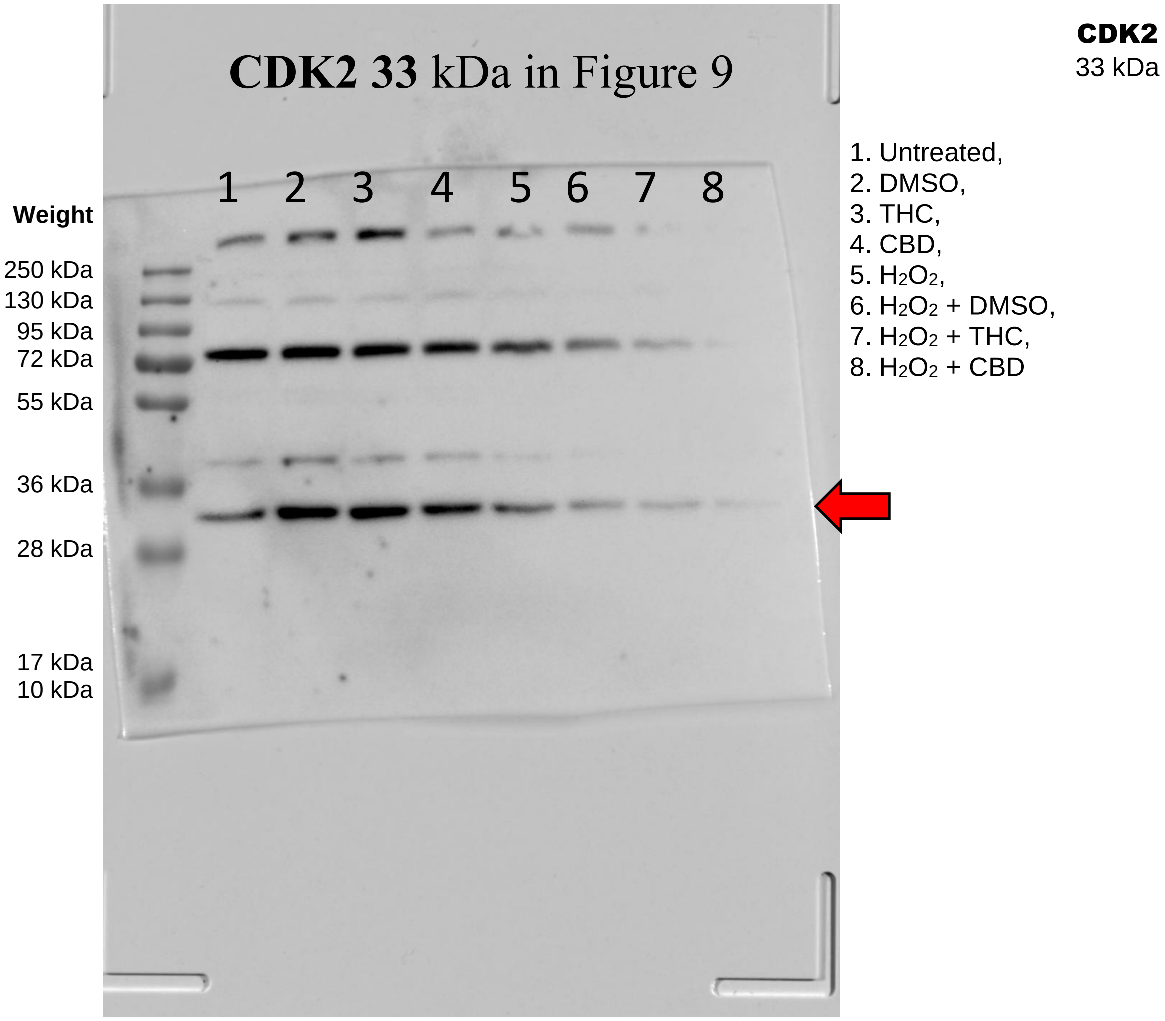


***Figure S12.*** Original Western blots of CCD-1064k (PDL 24) proteins showing CDK2 (molecular weight is 33 kDa). Red arrow indicates bands shown in Figure 9. This blot has been stripped and re-probed.

# **PCNA** 36 kDa in Figure 9

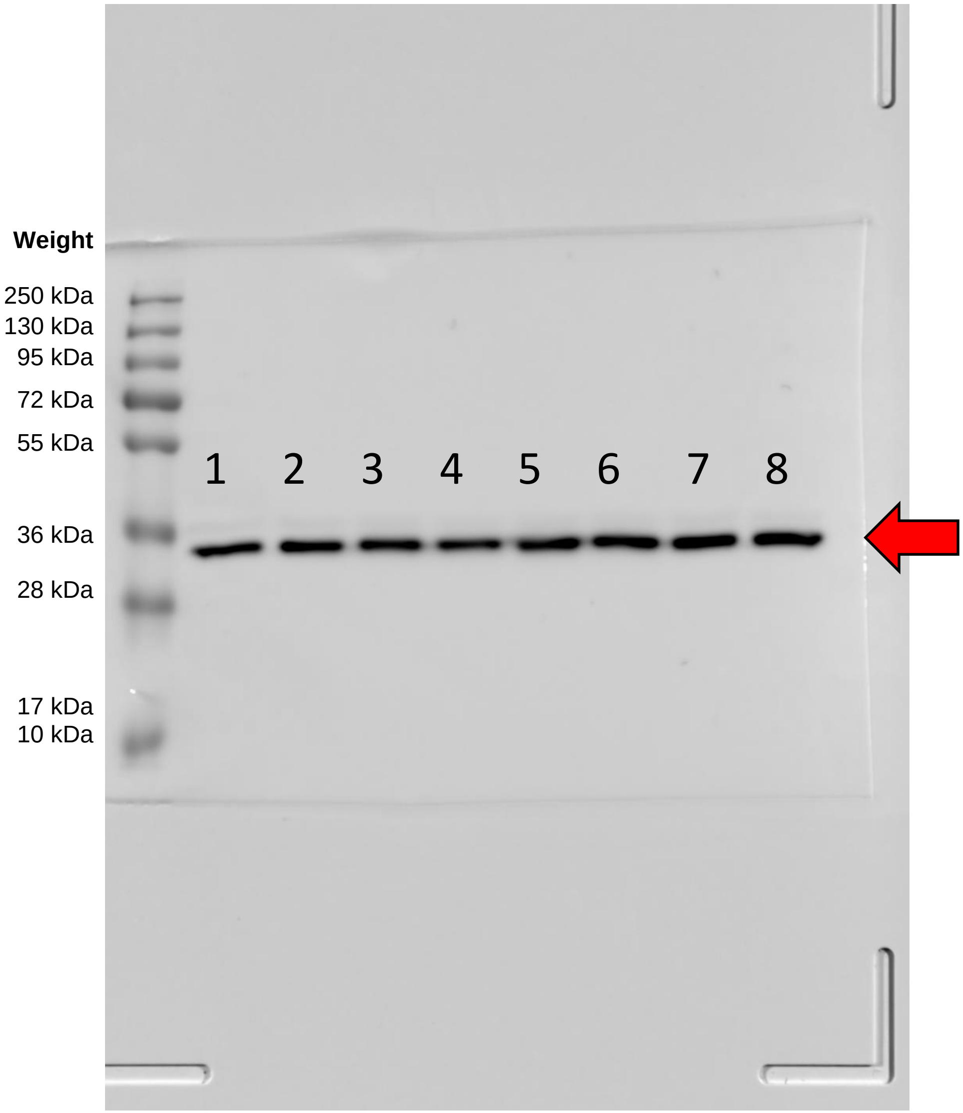


**PCNA**
36 kDa

1. 1. Untreated,
2. DMSO,
3. THC,
4. CBD,
5. $H_2O_2$,
6. $H_2O_2$ + DMSO,
7. $H_2O_2$ + THC,
8. $H_2O_2$ + CBD

***Figure S13.*** Original Western blots of CCD-1064k (PDL 24) proteins showing PCNA (molecular weight is 36 kDa). Red arrow indicates bands shown in Figure 9.

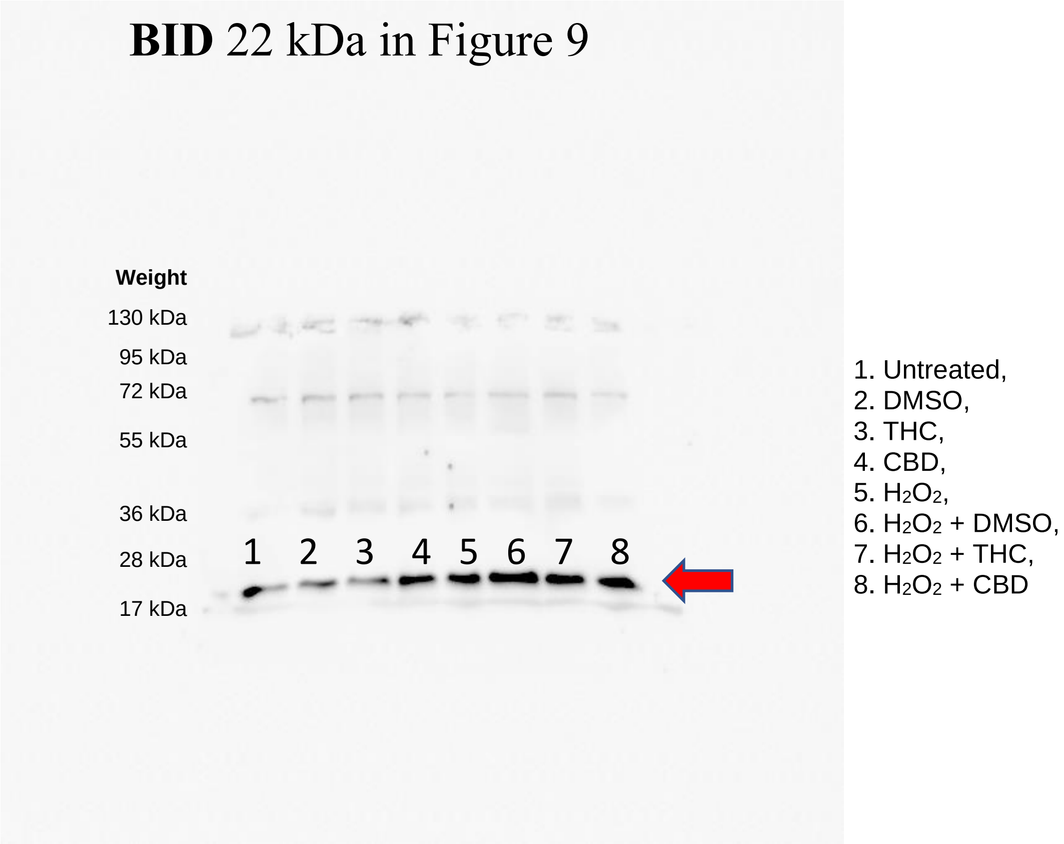


***Figure S14.*** Original Western blots of CCD-1064k (PDL 24) proteins showing BID, (molecular weight is 22 kDa). Red arrow indicates bands shown in Figure 9. This blot has been stripped and re-probed.

**p53** 53 kDa in Figure 9

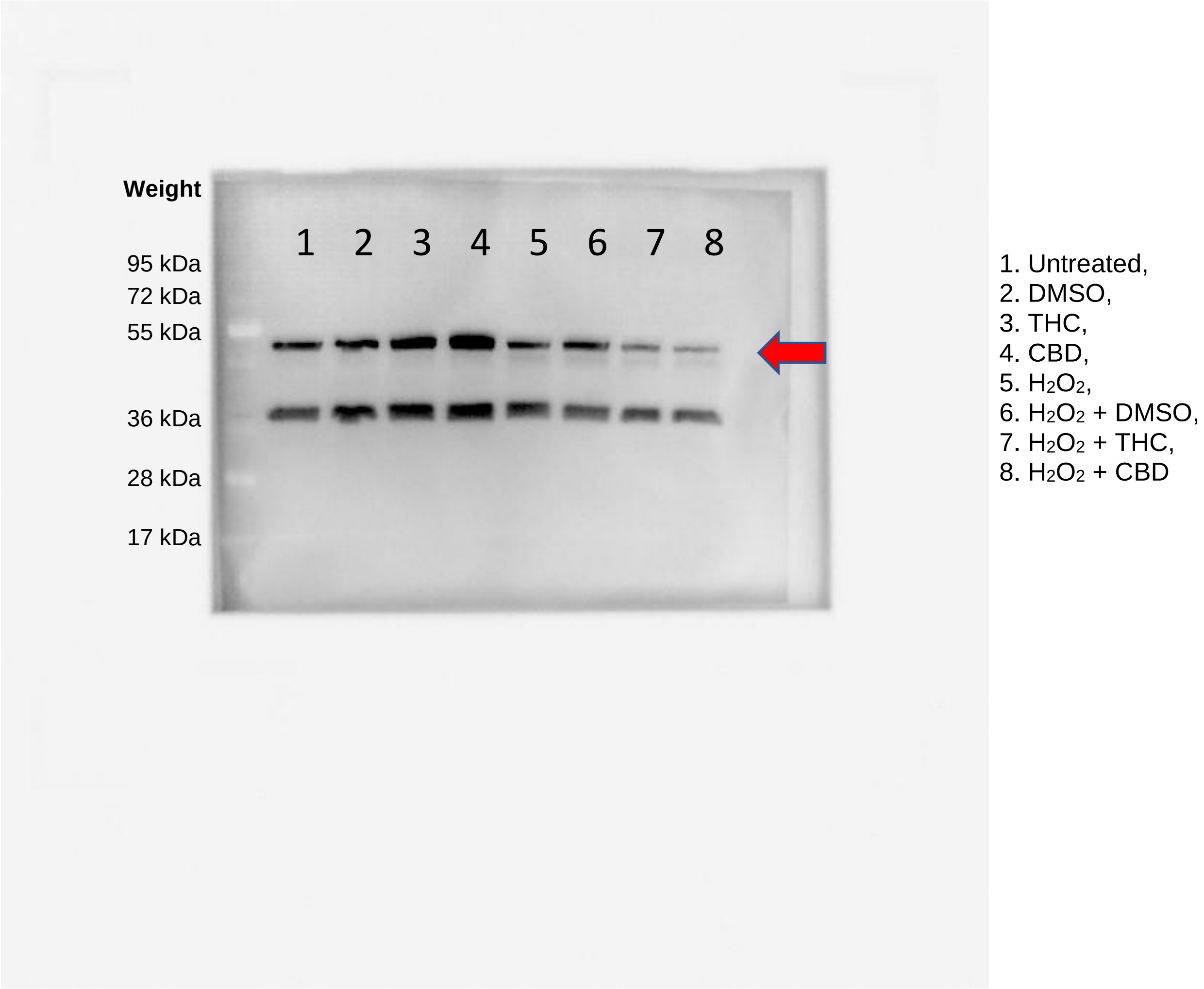


***Figure S15.*** Original Western blots of CCD-1064k (PDL 24) proteins showing p53, (molecular weight is 53 kDa). Red arrow indicates bands shown in Figure 9. This blot has been stripped and re-probed.

**p21** 21 kDa in Figure 9

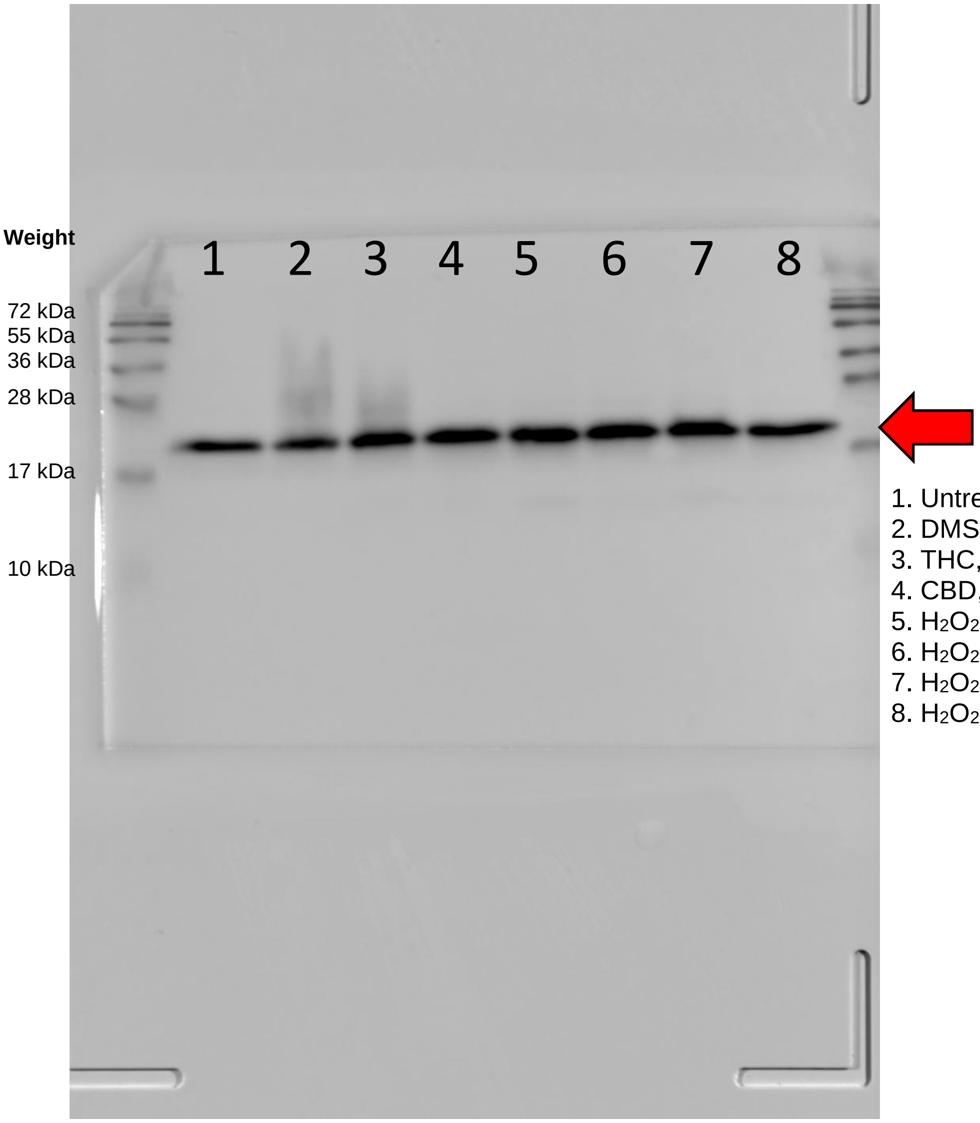


1. Untreated,
2. DMSO,
3. THC,
4. CBD,
5. $H_2O_2$,
6. $H_2O_2$ + DMSO,
7. $H_2O_2$ + THC,
8. $H_2O_2$ + CBD

***Figure S16.*** Original Western blots of CCD-1064k (PDL 24) proteins showing p21, (molecular weight is 21 kDa). Red arrow indicates bands shown in Figure 9.

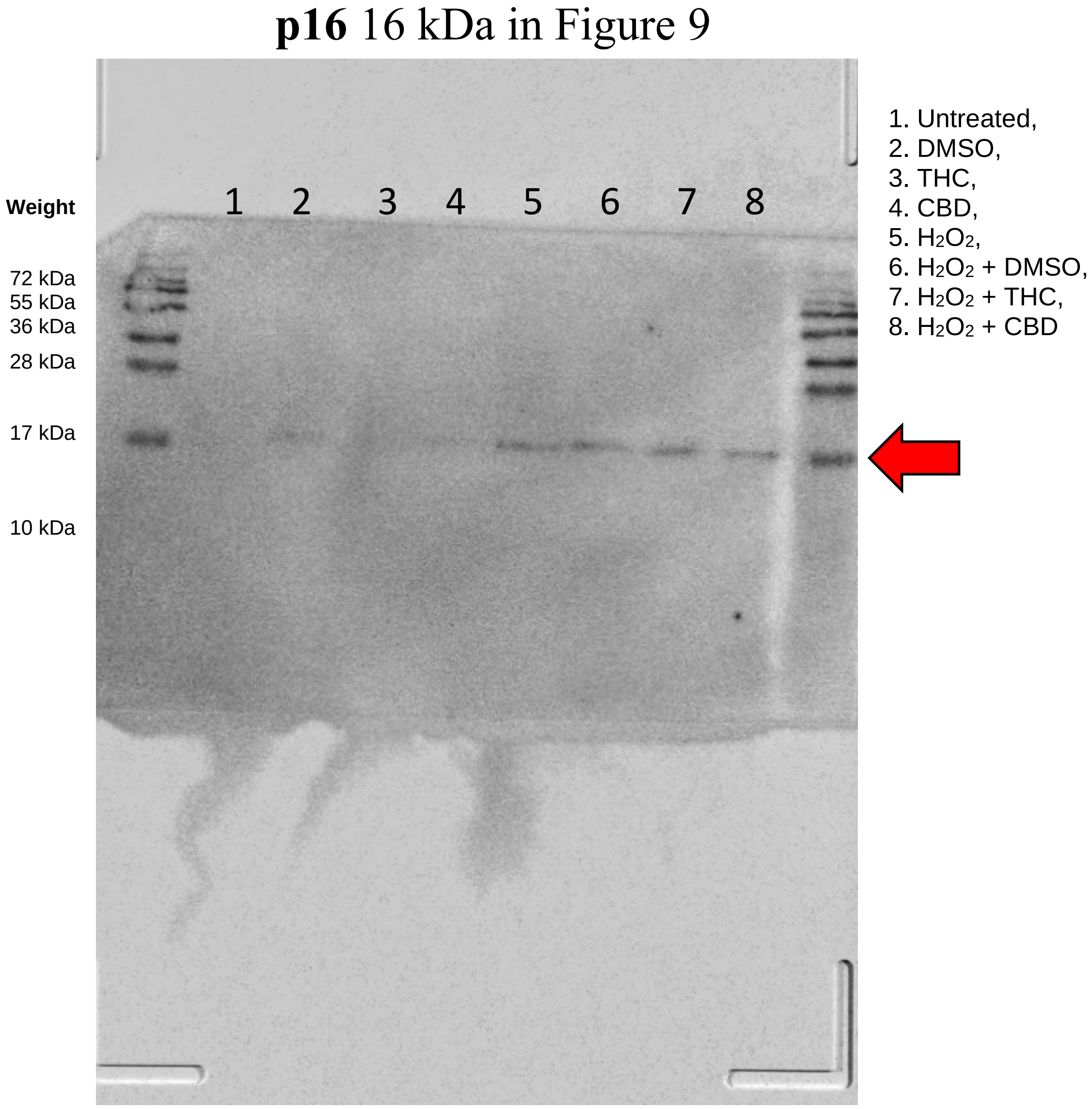


***Figure S17.*** Original Western blots of CCD-1064k (PDL 24) proteins showing p16, (molecular weight is 16 kDa). Red arrow indicates bands shown in Figure 9.

**NF-κB** 65 kDa in Figure 9

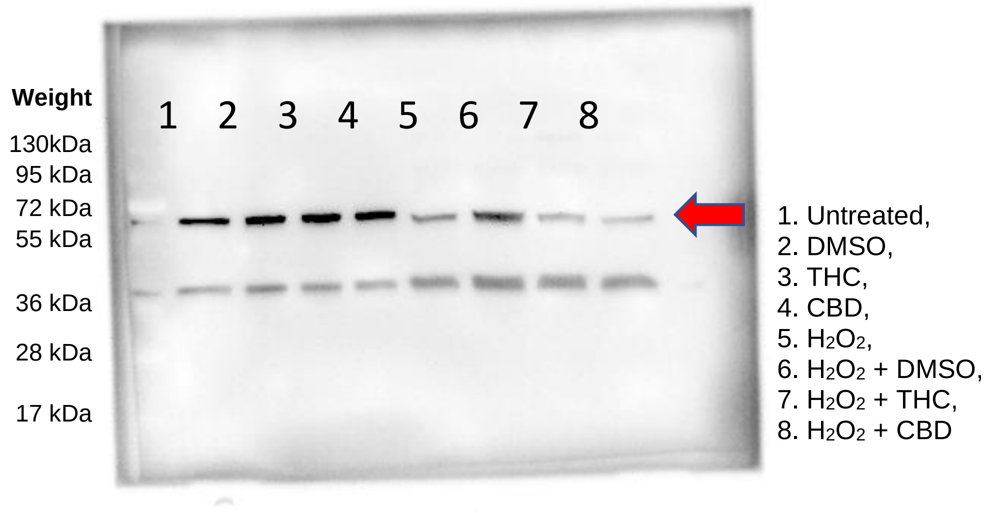


***Figure S18.*** Original Western blots of CCD-1064k (PDL 24) proteins showing NF-κB*,* (molecular weight is 65 kDa). Red arrow indicates bands shown in Figure 9. This blot has been stripped and re-probed.

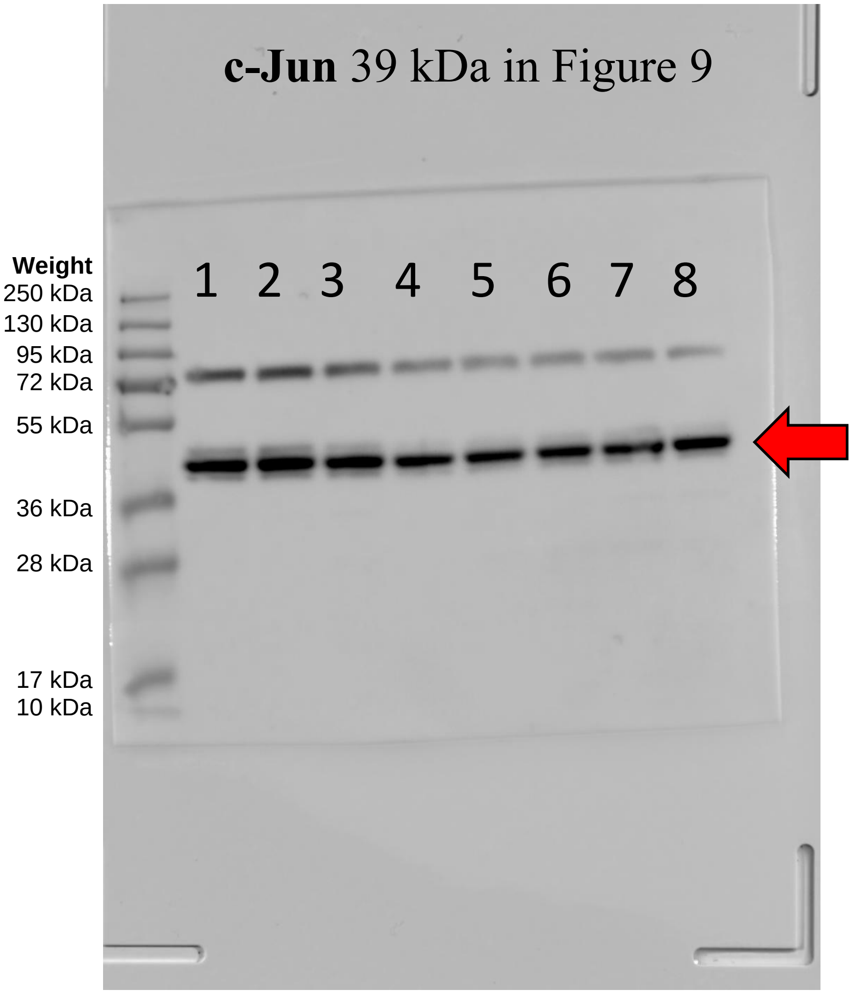


**c-Jun**
39 kDa

1. Untreated,
2. DMSO,
3. THC,
4. CBD,
5. $H_2O_2$,
6. $H_2O_2$ + DMSO,
7. $H_2O_2$ + THC,
8. $H_2O_2$ + CBD

***Figure S19.*** Original Western blots of CCD-1064k (PDL 24) proteins showing c-Jun, (molecular weight is 39 kDa). Bands shown in Figure 9. This blot has been stripped and re-probed.

**GAPDH** 36 kDa in Figure 9

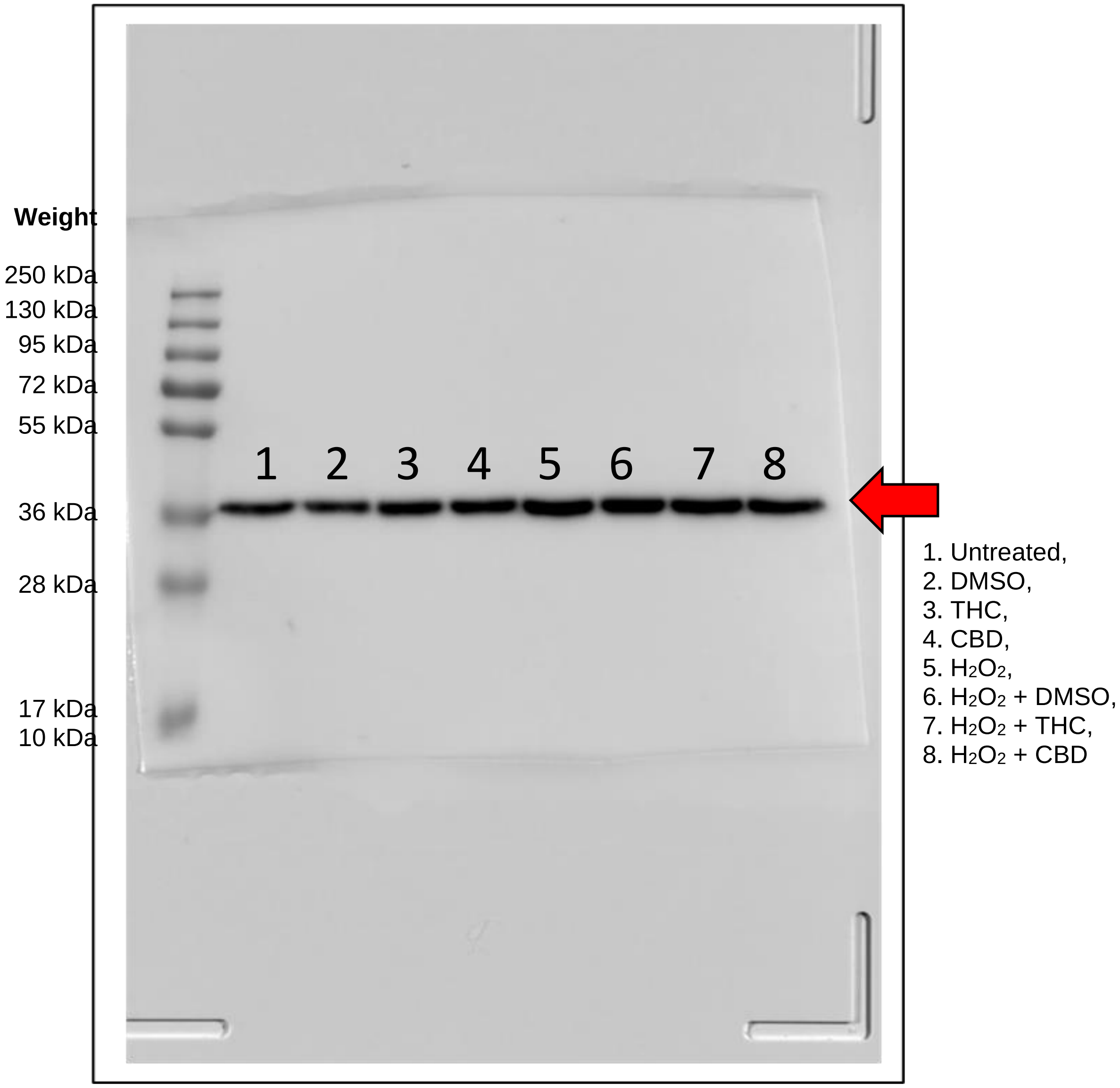


***Figure S20.*** Original Western blots of CCD-1064k (PDL 24) proteins showing GAPDH, (molecular weight is 36 kDa). Bands shown in Figure 9.

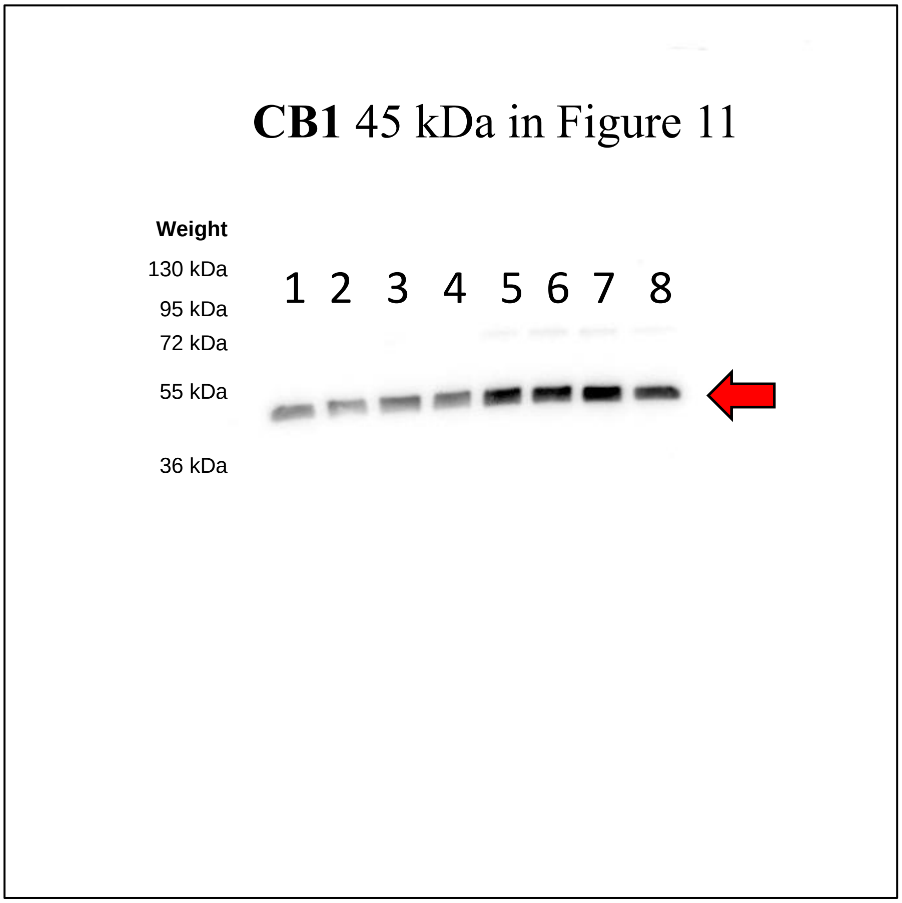


1. Untreated,
2. DMSO,
3. THC,
4. CBD,
5. $H_2O_2$,
6. $H_2O_2$ + DMSO,
7. $H_2O_2$ + THC,
8. $H_2O_2$ + CBD

***Figure S21.*** Original Western blots of CCD-1064k (PDL 24) proteins showing CB1, (molecular weight is 45 kDa). Bands shown in Figure 11. This blot has been stripped and re-probed.

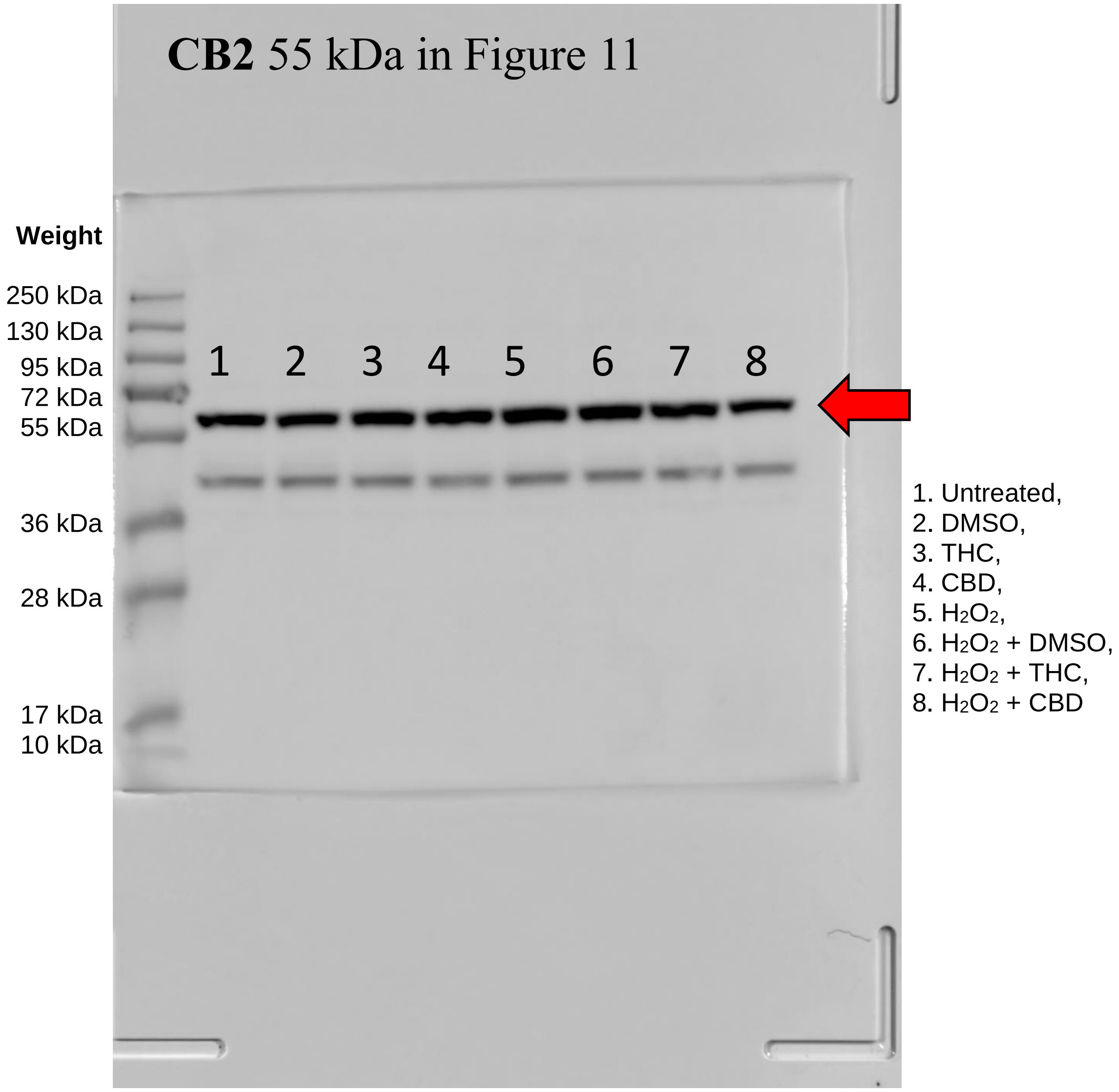


***Figure S22.*** Original Western blots of CCD-1064k (PDL 24) proteins showing CB2 (molecular weight is 55 kDa), Red arrow indicates bands shown in Figure 11. This blot has been stripped and re-probed.

SIRT1
120 kDa

# **SIRT1** 120 kDa in Figure 11

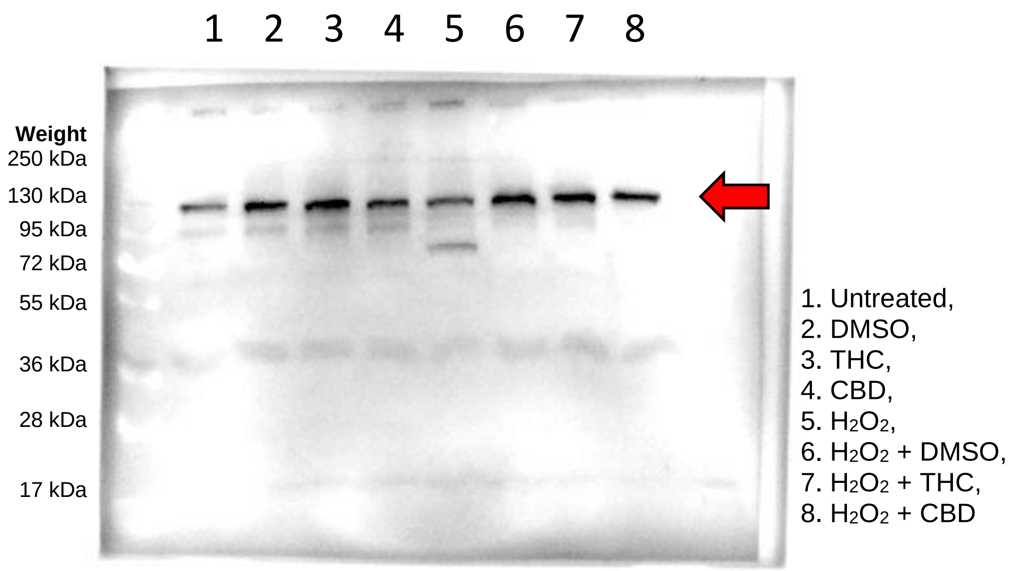


***Figure S23.*** Original Western blots of CCD-1064k (PDL 24) proteins showing SIRT1 (molecular weight is 120 kDa). Bands shown in Figure 11. This blot has been stripped and re-probed.

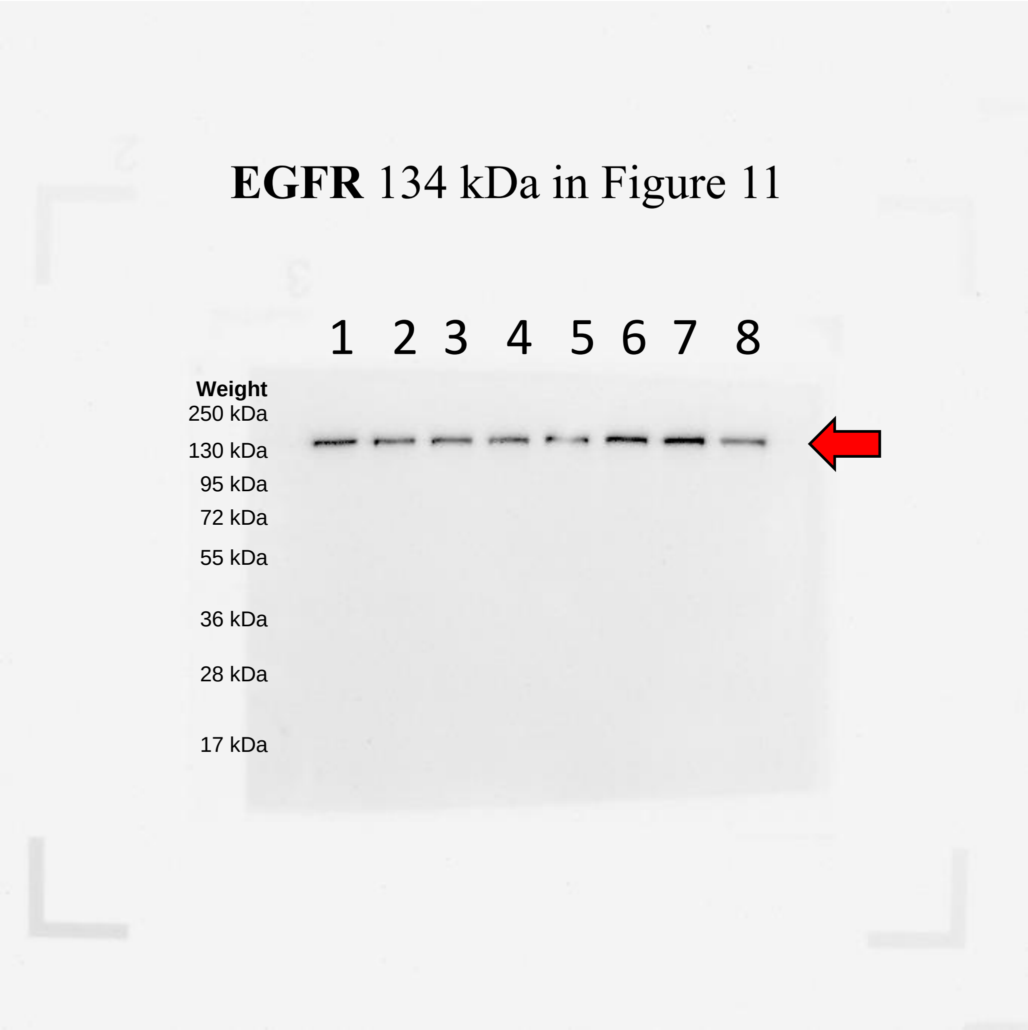


**EGFR**
134 kDa

1. Untreated,
2. DMSO,
3. THC,
4. CBD,
5. $H_2O_2$,
6. $H_2O_2$ + DMSO,
7. $H_2O_2$ + THC,
8. $H_2O_2$ + CBD

***Figure S24.*** Original Western blots of CCD-1064k (PDL 24) proteins showing EGFR (molecular weight is 134 kDa) Bands shown in Figure 11.

# **GAPDH** 36 kDa in Figure 11

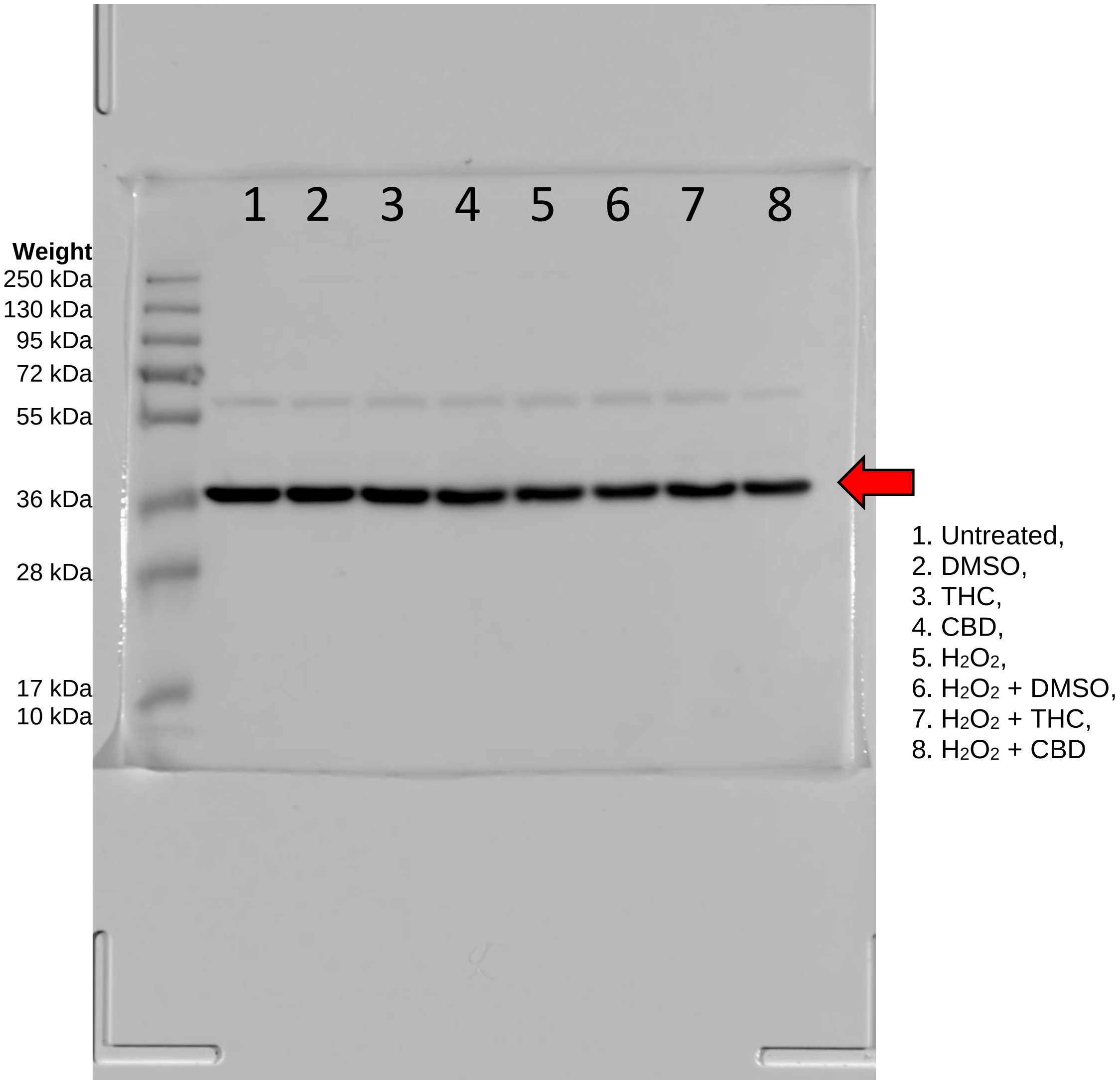


***Figure S25.*** Original Western blots of CCD-1064k (PDL 24) proteins showing GAPDH (molecular weight is 36 kDa). Bands shown in Figure 11. This blot has been stripped and re-probed.
